# Panel estimated Glomerular Filtration Rate (GFR): Statistical considerations for maximizing accuracy in diverse clinical populations

**DOI:** 10.1371/journal.pone.0313154

**Published:** 2024-12-02

**Authors:** Nora F. Fino, Lesley A. Inker, Tom Greene, Ogechi M. Adingwupu, Josef Coresh, Jesse Seegmiller, Michael G. Shlipak, Tazeen H. Jafar, Roberto Kalil, Veronica T. Costa e Silva, Vilmundur Gudnason, Andrew S. Levey, Ben Haaland

**Affiliations:** 1 Division of Biostatistics, Department of Population Health Sciences, University of Utah Health, Salt Lake City, Utah, United States of America; 2 Division of Nephrology, Department of Medicine, Tufts Medical Center, Boston, Massachusetts, United States of America; 3 Department of Epidemiology, John Hopkins Bloomberg School of Public Health, Baltimore, Maryland, United States of America; 4 Department of Laboratory Medicine and Pathology, University of Minnesota, Minneapolis, Minnesota, United States of America; 5 Kidney Health Research Collaborative, San Francisco Veterans Affair Medical Center and University of California, San Francisco, California, United States of America; 6 Program in Health Services and Systems Research, Duke-NUS Medical School, Singapore, Singapore; 7 Department of Medicine, Aga Khan University, Karachi, Pakistan; 8 Division of Nephrology, Department of Medicine, University of Maryland School of Medicine, Baltimore, Maryland, United States of America; 9 Serviço de Nefrologia, Instituto do Câncer do Estado de São Paulo, Faculdade de Medicina, Universidade de São Paulo, São Paulo, Brazil; 10 Laboratório de Investigação Médica (LIM) 16, Faculdade de Medicina da Universidade de São Paulo, São Paulo, SP, Brazil; 11 Faculty of Medicine, University of Iceland, Reykjavik, and the Icelandic Heart Association, Kopavogur, Iceland; University of Udine, ITALY

## Abstract

Assessing glomerular filtration rate (GFR) is critical for diagnosis, staging, and management of kidney disease. However, accuracy of estimated GFR (eGFR) is limited by large errors (>30% error present in >10–50% of patients), adversely impacting patient care. Errors often result from variation across populations of non-GFR determinants affecting the filtration markers used to estimate GFR. We hypothesized that combining multiple filtration markers with non-overlapping non-GFR determinants into a panel GFR could improve eGFR accuracy, extending current recognition that adding cystatin C to serum creatinine improves accuracy. Non-GFR determinants of markers can affect the accuracy of eGFR in two ways: first, increased variability in the non-GFR determinants of some filtration markers among application populations compared to the development population may result in outlying values for those markers. Second, systematic differences in the non-GFR determinants of some markers between application and development populations can lead to biased estimates in the application populations. Here, we propose and evaluate methods for estimating GFR based on multiple markers in applications with potentially higher rates of outlying predictors than in development data. We apply transfer learning to address systematic differences between application and development populations. We evaluated a panel of 8 markers (5 metabolites and 3 low molecular weight proteins) in 3,554 participants from 9 studies. Results show that contamination in two strongly predictive markers can increase imprecision by more than two-fold, but outlier identification with robust estimation can restore precision nearly fully to uncontaminated data. Furthermore, transfer learning can yield similar results with even modest training set sample size. Combining both approaches addresses both sources of error in GFR estimates. Once the laboratory challenge of developing a validated targeted assay for additional metabolites is overcome, these methods can inform the use of a panel eGFR across diverse clinical settings, ensuring accuracy despite differing non-GFR determinants.

## Introduction

Accurate assessment of glomerular filtration rate (GFR) is critical for patient care, especially for those with chronic diseases. Chronic kidney disease (CKD) affects approximately 9% of the world’s population (697 million people) and GFR estimation from serum creatinine is recommended and used globally [1]. CKD is common in patients with other chronic diseases, increases the risk for adverse outcomes for such diseases, and impacts on the decisions regarding their management and treatment. For example, approximately 20–50% of individuals diagnosed with heart or liver disease also have CKD [2–5]. The presence of CKD increases the risk of mortality by almost two-fold in heart failure patients [6] and a seven-fold increase in cirrhosis patients [3]. Many of the medications used to treat these diseases are cleared by the kidney, or are toxic to it, requiring assessment of the level of GFR prior to dosing. Standard practice for assessing kidney function in adults [1, 7, 8] is to estimate GFR (eGFR) using the Chronic Kidney Disease Epidemiology Collaboration (CKD-EPI) equations [9], which combine the endogenous filtration markers creatinine and/or cystatin-c as measured in blood with sex and age. Creatinine is measured as part of a basic metabolic panel and eGFR using creatinine (eGFRcr) is reported by clinical laboratories, allowing eGFR to be used for all routine decisions [10]. eGFR using both creatinine and cystatin-C (eGFRcr-cys) is generally more accurate than either GFRcr or eGFRcys and is used as a confirmatory test when eGFRcr is not thought to be accurate and the clinical decision is based on the level of GFR. [1]

In principle, when equations developed in one population are applied to a new population, observed differences in eGFR compared to measured GFR (mGFR) are predominantly due to the presence of non-GFR factors that affect the serum concentration of endogenous filtration markers (non-GFR determinants) [11–13]. Non-GFR determinants include generation, renal tubular reabsorption and secretion, and extra-renal elimination of the filtration markers. The CKD-EPI equations were developed in diverse populations with and without CKD and with respect to many key factors such as the level of GFR, diabetes, and age, but did not include populations with other chronic diseases. Thus, while these equations have been demonstrated to be reasonably accurate in general or CKD populations, they are less accurate in populations with other chronic diseases. For example, in patients with heart failure or cirrhosis, eGFR errors of over 30% can occur in 35–60% of cases [14–16], compared to roughly 15% in the development populations [9]. Non-GFR determinants of filtration markers may affect GFR estimation in two ways: First, increased variability in the distributions of non-GFR determinants in the application population relative to the development population may produce greater imprecision of the eGFR relative to mGFR in application populations. Second, systematic differences in the distributions of non-GFR determinants between application populations and the development populations may produce systematic differences in the relationship between mGFR and filtration markers, leading to biased estimation of GFR in the application populations.

We have hypothesized that the greater accuracy of eGFRcr-cys is due to non-overlapping non-GFR determinants of these two markers [17]. For example, eGFRcr is overestimated in patients with muscle wasting, and eGFRcys is underestimated in patients with higher levels of adiposity, smoking and chronic inflammation [18]. Thus, using both markers in eGFRcr-cys in theory balances the opposite effects of these non-GFR factors which are common in people with chronic disease. However, this balance cannot be expected to be present in all settings. For example, some medications may affect kidney tubular secretion or extra-renal elimination of creatinine, but these same medications are not known to affect the level of cystatin-C [19–21]. In these cases, eGFRcys may be more accurate than eGFRcr-cys. It is not possible for a physician to be able to appreciate the presence and magnitude of the non-GFR determinants of each filtration marker in an individual patient, leading to uncertainty as to which eGFR to use. To overcome this central challenge of GFR estimation, we hypothesized that a larger number of endogenous filtration markers (here on referred to as panel eGFR) will be required [11, 13, 22]. Ideally, by incorporating additional markers with minimally overlapping non-GFR determinants, a panel eGFR could diminish the importance of any single marker. However, methods for robust prediction within application populations will be required.

We note that previous statistical literature on robust prediction has focused on reducing the effect of outliers *within the development data set*. [23] These methods, such as least trimmed squares [24], MM regression [25, 26], robust model selection [27], and robust partial least squares [28] typically estimate robust model coefficients by down-weighting large residuals within the development data such that outliers do not heavily influence the model coefficients. Multivariate methods, such as robust principal components [17, 21], can also be used to identify outliers within the development data. However, to our knowledge, the question of how predictive models constructed from a development population can be modified to reduce the impact of an increased rate of outliers *in an application population* has yet to be addressed. Separately, the literature also has many examples [29–32] of redeveloping equations in new applications, especially geographic regions, to overcome systematic differences in distributions of non-GFR determinants between the application population and the population contributing to the development data sets. However, the cost of obtaining an adequate number of participants with mGFR to estimate separate equations in each target population with sufficient accuracy and robustness for clinical use is likely to be prohibitive. Here, the application of transfer learning may be useful to tailor estimates for specific patient applications.

In this analysis, we evaluated a panel of eight markers (5 metabolites including creatinine and 3 low molecular weight proteins including cystatin-C) in 3,554 participants from 9 studies representing diverse patient populations. In separate investigations, we are in the process of identifying additional metabolites to be included in a metabolite panel eGFR [22]. This new panel is intended as an independent complementary test for clinical settings in which eGFRcr-cys is not sufficiently accurate for the medical decision-making. Critical to this use are methods that can robustly estimate GFR in new applications, namely, methods that de-emphasize outlying markers within new individuals and tailor estimates to specific groups. The goal of this paper is to propose and evaluate methods for estimating GFR based on multiple filtration markers in application populations with potentially higher rates of outlying predictors than in the development data. We further apply transfer learning techniques to account for systematic differences between the application population and development populations. We demonstrate these methods using our existing data as a proof-of-concept, but we intend this work to be applied to the panel under development [22]. We expect that certain applications may have higher rates of outlying predictor values of the markers compared to our current datasets; therefore, we simulated these applications by altering the relative frequencies of outliers in application datasets.

## Methods

### Data

We used data from the CKD-EPI [33] research database, which consists of data from diverse research studies and clinical populations with GFR measured via urinary or plasma clearance of exogenous filtration markers (measured GFR, or mGFR, **S1 Table**). In the current analysis, we included all studies in which targeted assays were available for eight filtration markers, including five metabolites [creatinine (mg/dL), acetylthreonine (μg/mL), pseudouridine (μg/mL), tryptophan (μg/mL), and phenylacetylglutamine (μg/mL)] and three low molecular weight proteins (LMWP) [cystatin-C (mg/L), beta-2-microglobulin (B2M, mg/L), and beta trace protein (BTP, mg/L)] [13, 34]. Metabolites were measured using ultra-performance liquid chromatography–tandem mass spectrometry (UPLC-MS/MS) [34]. LMWP were measured using immunoturbidimetric (B2M and cystatin-c) or immunonephelometric (BTP) assays [35]. We adjusted mGFR using iohexol and inulin clearance to align with iothalamate by increasing values by five percent, as done previously [9, 35, 36] To stabilize variability in residual errors, we log-transformed mGFR and all predictors and included all observations with values for mGFR and all eight predictors, excluding 211 participants (5.6%) with missing data.

### Statistical methods

**Fig 1:**
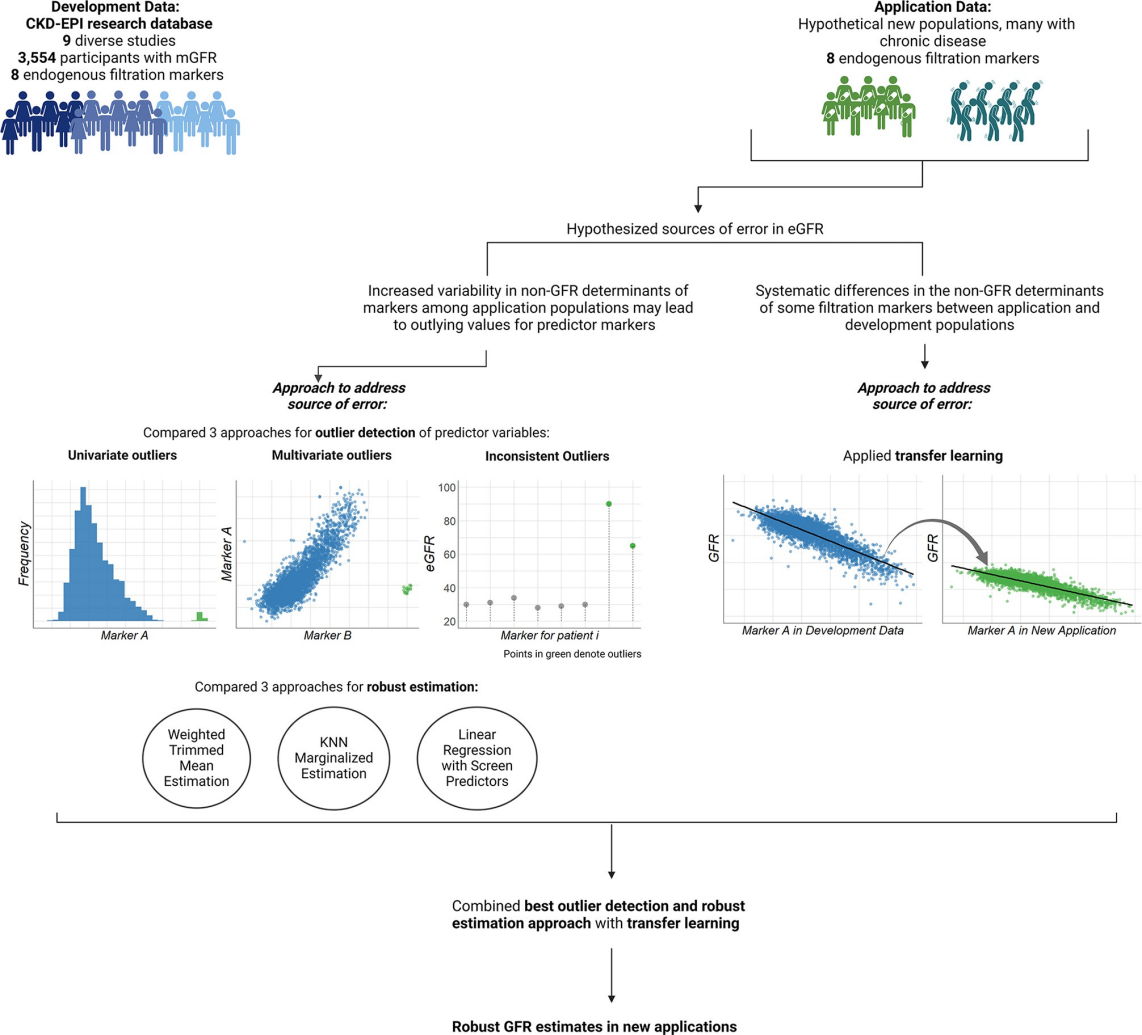
Schematic overview of our approach to estimate GFR robustly in new applications. Application of GFR estimating equations that was developed in one population (blue figures on the top left) will be less accurate in different populations (green figures on the top right) due to variation in the distribution of factors that determine the level of the filtration markers other than GFR (referred to as non GFR determinants). Non-GFR determinants of markers can affect GFR estimation accuracy by distorting markers in individual patients (plots on the left hand side) and causing systematic differences between development and application populations (plots on the right hand side). We propose methods to address both errors, using techniques to detect outlying predictor variables, robust estimation, and transfer learning. Finally, we combine these approaches for robust GFR estimates in new applications.

Our overall goal was to develop methods to estimate GFR from multiple markers while considering alterations in non-GFR determinants of the markers in new applications (**Fig 1**). First, we combined three approaches for detecting outlying predictors in application data with robust estimation methods, enabling us to evaluate nine distinct approaches for mGFR estimation (**Table 1**). Next, because our current development datasets do not include many patients with acute illnesses, these datasets would likely exhibit fewer outlying predictors than would be seen in real-world experiences. Therefore, we evaluated these approaches using statistical simulation to emulate application scenarios with increased outlying predictors. Finally, we evaluated the possible merits of transfer learning in estimating GFR. We then integrated both methods (outlier detection with robust prediction and transfer learning) to customize equations for distinct patient groups.

**Table 1:** Summary of each combination of outlier detection methods and robust estimation approaches. We combined each outlier detection method with each estimation approach such that there were nine different appoaches for robust GFR estimation in new application data.

	Outlier Detection Method			
Robust Estimation Approach	Univariate Outliers	Multivariate Outliers	Inconsistent Outliers
**Weighted Trimmed Mean Estimation** ^**a**^	Omit univariate outliers,	Shrink multivariate outliers using Winsorization,	Omit two most inconsistent predictors,	
	Calculate eGFR using weighted mean of univariate model estimates			
		Calculate eGFR using weighted mean of univariate model estimates	Calculate eGFR using weighted mean of univariate model estimates	
**KNN Marginalized Estimation**	Omit univariate outliers,	Shrink multivariate outliers using Winsorization,	Omit two most inconsistent predictors,	
	Calculate eGFR using linear regression with 8 predictors (no univariate outliers) or KNN marginalized estimation (univariate outliers present).	Calculate eGFR using linear regression using all 8 predictors.	Calculate eGFR KNN marginalized estimation using the most consistent 6 markers	
**Linear Regression with Screened Predictors**	Omit univariate outliers,	Shrink multivariate outliers using Winsorization,	Omit two most inconsistent predictors,	
	Calculate eGFR using multivariable linear regression using all markers not identified univariate outlier.	Calculate eGFR using calculate eGFR using multivariable linear regression	Calculate eGFR using multivariable linear regression of the most consistent 6 markers	

^a^Weight univariate estimates by corresponding metabolite’s correlation with mGFR

In descriptive analysis, we present the average within-study correlations of the markers with mGFR across studies. We fit single-marker and multi-marker (including all eight markers) regression models for mGFR separately for each study, and assessed predictive accuracy using root mean square error (RMSE), with lower values indicating better accuracy. To assess how predictive accuracy changes by including more markers in this data, we fit ordinary least squares linear regression models for all possible collections of the eight markers (i.e., all models containing a single marker, all models containing two markers, etc.). Application data error rates were approximated through ten iterations of ten-fold cross-validation.

### Outlier detection methods and robust estimation approaches

First, we compared three approaches to identify outlying predictors in the application data: (1) univariate outliers with respect to development populations, (2) multivariate outliers with respect to development populations, and (3) inconsistencies amongst the predictor markers (**Fig 1**). These approaches are described below:

1. *Univariate outliers with respect to development populations*: We identified individual filtration markers in the application data that were outliers with respect to the univariate distributions of the development data. Specifically, we defined outliers in the application data as those values more extreme than the 1^st^ or 99^th^ percentiles of the predictor in the development data. We then based GFR estimates on markers not identified as univariate outliers.
2. *Multivariate outliers with respect to development populations*:: We recognized that outliers may only be visible when examining joint distributions of the markers. Using the data-cleaning approach outlined in Khan et. al [27]. We computed the squared Mahalanobis distance *D_i_* of the *ith* subject in the new application data *X_test_* from the mean of the development data. Then, for points with large distances, we Winsorized, or shrank, their values to the boundary of a tolerance ellipse based of the development data *X_train_*. Specifically, for all observations, we replaced the *ith* row in *X_test_* with the transformation $U_{i}=\min\left(\sqrt{\frac{c}{D_{i}}},1\right)*{X_{test}}_{i}$, where *c* is the 99^th^ percentile of a chi-squared distribution with 8 degrees of freedom, *χ*^2^(0.99, *p* = 8). A hypothetical example of this method applied to the application data is shown in**S1 Fig**. The Winsorized data with no problematic predictors was then used in estimates in the application data. It is worth noting that for the multivariate outlier approach, outlying points are shrunken before the estimation step and all eight (cleaned) predictors are used in the estimation methods described below (i.e., the specific steps for problematic points in the robust estimation steps are not applied).
3. *Inconsistencies among the predictor markers*: We considered when GFR estimates from individual markers in the application data are inconsistent. For example, most markers may predict low GFR while one or two markers might predict high GFR. To identify the inconsistent markers, we fit eight single-marker linear regressions in the development data and calculated an eGFR using each univariate model in the application data. We then choose the consistent markers as the collection of six markers with the smallest sum of all absolute pairwise differences in their univariate GFR estimates.

Next, we integrated each outlier detection method with approaches for robust prediction. In general, we compared approaches to GFR estimation when eGFR estimates in new application data are based solely on non-outlying predictors. These approaches are given by:

1. *Weighted trimmed mean estimation*: We fit eight single-marker linear models to the development data and computed estimates using each univariate model on the application data. Our final GFR estimate was calculated as the weighted mean of the estimates based on non-outlying predictors, defining using each marker’s weight as the absolute value of its pairwise correlation with mGFR. Weighting by each marker’s correlation with mGFR is motivated by partial least squares [37].
2. *Linear regression and K-nearest neighbors (KNN) marginalized estimation*: We used the development dataset to fit a linear model with all eight predictors. For observations in the application data with no problematic predictors, we used this model to predict their mGFR. For observations in the application dataset with problematic predictors, we averaged linear regression estimates over a small collection of K-nearest neighbors of the non-outlying predictors in the development data. Ten random iterations of cross-validation within the development data were used to choose the optimal number of neighbors k.

1. *Linear Regression with Screened Predictors*: For each individual in the application data, we estimated GFR using a linear regression model fit on the development data that contains all the non-outlying predictors for this individual. When all subjects in the application data are treated as having two outlying predictors, and GFR estimates are based solely on a given individual’s six most consistent markers.

### Evaluation of approaches

We compared all approaches in terms of RMSE and average log bias (calculated as $\log\;mGFR-\log\;\hat{eGFR}$, such that positive values indicate underestimates of mGFR and negative values indicate overestimates of mGFR. Exponentiated values of bias on the log scale can be interpreted as the ratio of mGFR to eGFR.

As a reference to when no special attention is paid to problematic predictors, we also fit a traditional ordinary least squares linear regression using all markers in the development data and applied this model to the application data. In all approaches, we performed ten random iterations of ten-fold cross-validation to approximate errors in new populations.

### Simulation of application data

As mentioned above, we anticipated that our current development would exhibit fewer outlying predictors. As a result, we employed statistical simulations to mimic scenarios where the occurrence of outlying predictors is more frequent. We used a multi-step processto add these outlying predictor variables, or simulated contamination, to the test datasets in the cross-validation. In each iteration of the cross-validation, a random 10% of the test observations were substantially altered by replacing one or more of the individual observed markers with outliers using a procedure outlined in the S2 File.

We compared three different types of contamination: mean contamination, variance contamination and both mean and variance contamination. An example of each contamination model is shown in **S2 Fig.**

### Transfer learning

We posited that equations may still need to be tailored to specific application populations due to systematic alterations in the relationship of the markers and GFR to achieve optimal accuracy. Transfer learning models could be useful in this situation as they leverage existing data, as well as the new application population. In general, construction of accurate estimation models requires a substantial amount of data, but in some cases, the available development data in the specific application population of interest may be restricted. However, we might have some richer data for the new application that is related to, but distinct from, the limited original data. In this case, we could borrow, or transfer, information from related tasks to help with the application. In the transfer learning literature, the specific application population is referred to as the target population, and the related populations are referred to as source populations. Broadly, we can train a model on a pooled source population, then use a small target dataset to tailor the model for the target population. A major benefit is that often the target dataset does not need to be large to achieve good accuracy in the greater target population of interest.

We hypothesize that there still may be systematic shifts beyond outliers in the current pooled dataset of multiple studies. As an assessment of the merits of transfer learning, we used each study as the target population and the remaining studies as source populations. For 10 iterations, we split the target data into cross-validation training and test sets. We fit transfer learning regression for generalized linear models [38] using the source populations and training target data, and used the fitted model to compute RMSE and bias on test target data. Briefly, this algorithm employs a two-step transfer: first, all source data is pooled to obtain a rough estimate of the coefficients. Then, target data is used to correct any bias in the pooled estimator. A small internal three-fold cross-validation step is used within the algorithm to determine which source populations are transferable.

We varied our cross-validation split to contain various portions of the target data and computed the average RMSE and bias across each iteration. We compared the transfer learning models to linear regression models fit using all studies except for a single held-out study used as the application dataset, as well as study-specific linear regression models trained with various proportions of the target observations and applied to the remaining observations.

Lastly, we performed an assessment of the combination of transfer learning models with outlier detection and robust prediction when applied to contaminated data. For each study, we fit linear models that trained on a random sample of 25 observations and tested on the remaining observations with no added contamination to be used as a benchmark for comparison. We then added mean and variance contamination to a single excellent predictor (pseudouridine alone) or to two excellent predictors (pseudouridine and cystain-C), and compared to linear remodels developed and applied within the given study, linear models developed and applied within the given study but with outlier identification and robust estimation, and finally transfer learning models for a given study with outlier identification and robust estimation. Finally, we repeated this analysis using a random sample of 50 and 100 (instead of 25) observations for the development data.

## Results

The study populations (N = 3,554) include participants with and without cancer, cardiovascular disease, and CKD across a wide range of ages, geographic locations, and race groups **(Table 2)**. Mean ages ranged from 35.4 (standard deviation (SD) 7.6) years in the Consortium for Radiologic Imaging Studies of Polycystic Kidney Disease (CRISP) to 80.2 (3.9) years in Age, Gene/Environment Susceptibility)-Reykjavik Study (AGES). The proportion of black individuals varied across the cohorts, spanning from 0% in AGES and Pakistan to 100% in African American Study of Kidney Disease and Hypertension (AASK). Average mGFR across cohorts varied, with mean values of 33.0 (11.6) mL/min/1.73m^2^ in the Modification of Diet in Renal Disease (MDRD) study to 96.1 (14.6) in Assessing Long Term Outcomes in Living Kidney Donors (ALTOLD).

**Table 2:** Summary of analytic dataset (N = 3,554).

	AASK	AGES	ALTOLD	Onco-GFR	CRISP	MDRD	MESA	Pakistan	UMN Donors
n	1027	573	129	288	55	484	192	518	288
Study Description	RCT of CKD progression	Cohort study of aging related diseases	Cohort study of kidney donor candidates	Cohort study of patients with solid tumors in Brazil	Cohort study of polycystic kidney disease	RCT of CKD progression	General population of older adults	General population of South Asians	Clinical Population of kidney donors
**Geography**	United States	Iceland	United States	Brazil	United States	United States	United States	Pakistan	United States
Age (years)	54.2 (10.5)	80.2 (3.9)	43.8 (11.4)	59.3 (13.2)	35.4 (7.6)	52.1 (11.9)	69.2 (8.6)	51.0 (10.1)	40.4 (12.5)
Black Race, n (%)	1027 (100.0)	0 (0.0)	3 (2.3)	39 (13.5)	1 (1.8)	36 (7.4)	91 (47.4)	0 (0.0)	10 (3.5)
Female Sex, n (%)	372 (36.2)	319 (55.7)	82 (63.6)	127 (44.1)	30 (54.5)	181 (37.4)	181 (37.4)	261 (50.4)	186 (64.6)
mGFR (ml/min/1.73m^2^)	56.8 (23.1)	62.8 (16.1)	96.1 (14.6)	77.2 (21.1)	87.4 (22.3)	33.0 (11.6)	78.6 (16.6)	88.3 (32.9)	93.8 (13.8)
Creatinine (mg/dL)	1.76 (0.88)	0.99 (0.39)	0.80 (0.14)	0.93 (0.37)	1.00 (0.22)	2.34 (0.94)	0.85 (0.18)	0.97 (0.79)	0.79 (0.15)
Cystatin-c (mg/L)	1.45 (0.60)	1.18 (0.38)	0.81 (0.12)	1.13 (0.36)	0.90 (0.18)	2.01 (0.58)	0.89 (0.15)	1.22 (0.72)	0.78 (0.11)
B2M (mg/L)	3.67 (2.46)	2.91 (1.35)	1.65 (0.23)	2.36 (0.95)	1.91 (0.60)	5.39 (2.34)	1.92 (0.42)	3.27 (2.86)	1.55 (0.27)
BTP (mg/L)	1.06 (0.99)	0.92 (0.42)	0.57 (0.09)	0.74 (0.38)	0.61 (0.18)	1.86 (0.72)	0.57 (0.15)	0.91 (0.89)	0.47 (0.10)
Acetylthreonine(μg/mL)	0.16 (0.09)	0.12 (0.05)	0.08 (0.01)	0.10 (0.04)	0.09 (0.02)	0.25 (0.13)	0.08 (0.01)	0.11 (0.10)	0.08 (0.01)
Phenylacetylglutamine (μg/mL)	1.47 (1.45)	1.46 (1.30)	0.48 (0.27)	0.98 (1.14)	0.62 (0.35)	2.61 (2.89)	0.84 (0.48)	1.05 (1.89)	0.51 (0.30)
Pseudouridine (μg/mL)	1.45 (0.81)	1.16 (0.55)	0.75 (0.10)	0.95 (0.37)	0.87 (0.18)	2.63 (1.46)	0.83 (0.15)	1.20 (1.11)	0.78 (0.11)
Tryptophan (μg/mL)	11.49 (2.89)	14.38 (2.45)	11.96 (2.26)	11.95 (2.53)	12.73 (2.84)	9.20 (2.23)	8.38 (1.83)	9.99 (2.14)	15.94 (2.68)

Data represents mean (standard deviation) unless specified otherwise. Further study details are provided in S1 Table.

Abbreviations: RCT: Randomized Controlled Trial; CKD: chronic kidney disease; mGFR: measured glomerular filtration rate; BTP: beta trace protein; B2M: beta-2-microglobulin; AASK: African American Study of Kidney Disease and Hypertension; AGES: Age, Gene/Environment Susceptibility)-Reykjavik Study; ALTOLD: Assessing Long Term Outcomes in Living Kidney Donors; Onco-GFR: oncology GFR; CRISP: Consortium for Radiologic Imaging Studies of Polycystic Kidney Disease; MDRD: The Modification of Diet in Renal Disease study; MESA, Multi-Ethnic Study of Atherosclerosis

**Fig 2:**
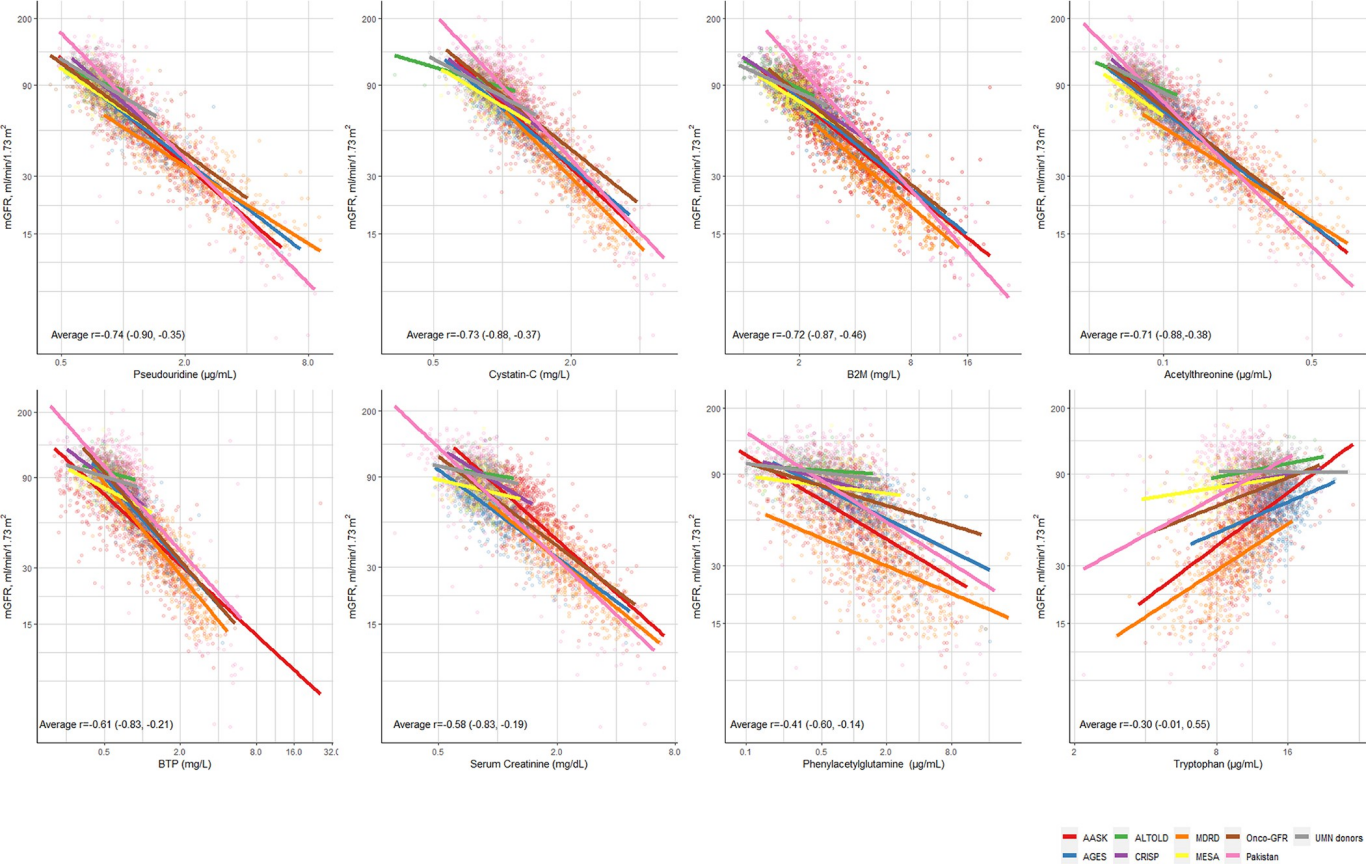
Regression models for mGFR using each marker by study. The average, minimum, and maximum correlations [Average r (minimum, maximum)] of each marker with mGFR across studies is provided within each plot.

The individual filtration markers varied both in their association with mGFR and their consistency across studies (**Fig 2)**. Five of the markers had stronger average Pearson correlations with mGFR across study than that of creatinine (r = -0.58): pseudouridine (r = -0.74), cystatin-C (r = -0.73), B2M (r = -0.72), acetylthreonine (r = -0.71), and BTP (r = -0.61), whereas phenylacetylglutamine and tryptophan had weaker correlations with mGFR than creatinine at -0.41 and 0.30, respectively. Estimated regression lines were generally similar by study for pseudouridine and cystatin-C, but considerably different for phenylacetylglutamine and tryptophan. **S2 and S3 Tables** present both single-marker and multi-marker linear regression models for mGFR by study. Notably, there is observable variability in the multivariable coefficients across different studies. For example, the multivariable coefficient for cystatin-C ranged from 0.00 in Pakistan to -0.37 in MDRD. Similarly, the multivariable coefficient for pseudouridine was -0.05 in Onco-GFR and -0.71 in Pakistan. R^2^ ranged from 0.86 in AGES to 0.37 in ALTOLD.

**S3 Fig** shows cross-validated RMSEs for all possible regression models of the eight markers. The 28 models containing six markers are able to predict mGFR almost as accurately as the single model with eight markers: cross-validated RMSEs ranged from 0.186 to 0.196 in models containing six markers, with 89.8–91.1% of individuals within 30% of their mGFR in cross-validation, compared to a cross-validated RMSE of 0.185 and 90.8% of individuals within 30% of their mGFR when using all eight markers. Therefore, if up to two markers are outlying, we can still make almost as accurate GFR estimates from the remaining six markers.

With no added contamination and application of the outlier detection and robust estimation approaches (**S4 Fig**), RMSEs were similar. The RMSE was smallest using the multivariate outlier approach with KNN marginalized estimation (0.182) and the univariate outlier approach with linear regression with screen predictors estimation (0.189). In general, RMSEs were largest using weighted trimmed mean estimation (RMSEs 0.208–0.221).

**Fig 3:**
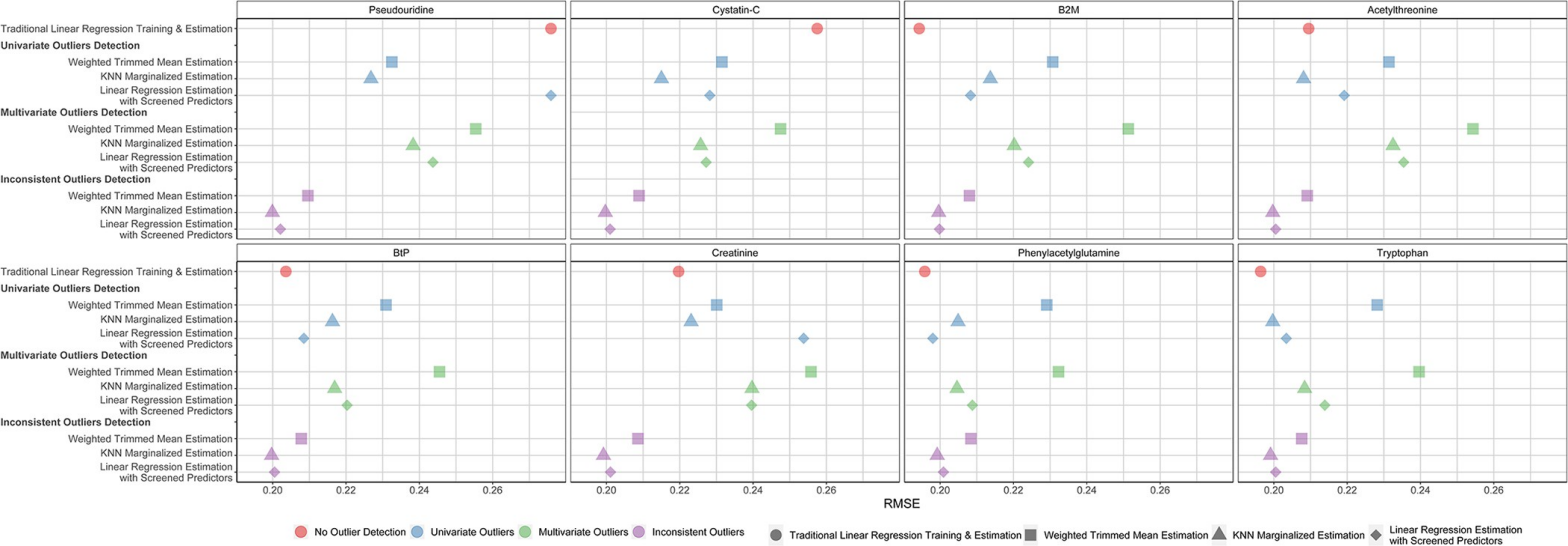
Comparison of RMSE using all modeling and prediction approaches after mean and variance contamination of each marker individually. The color of the points represents the underlying outlier detection strategy, and the shape represents the robust estimation approach. Results are averaged across ten cross-validation iterations. RMSE: Root Mean Square Error.

**Fig 3** displays RMSE for each outlier detection and robust estimation strategy after contaminating a single marker using mean and variance contamination. Given that the markers differ in their association with mGFR, we expected the impact of contamination on prediction to vary across the individual markers. Overall, contaminating markers that have stronger associations with mGFR, such as pseudouridine and cystatin-C, had a much larger impact on estimation error and bias than contaminating markers with weaker associations with mGFR, such as tryptophan. For example, when adding contamination to cystatin-C values in the test data but paying no special attention to outlying predictors, the cross-validated RMSE was 0.258. After identifying univariate outliers and applying the three robust estimation approaches, RMSEs improved to 0.215–0.232, but were still notably higher than linear regression with 8 markers and no added contamination (0.185). Similarly, using the multivariate outliers strategy, RMSEs ranged from 0.226–0.248. However, when removing inconsistent outliers, the RMSEs were very similar to the case when no contamination is present. For example, using linear regression with screened predictors, the RMSE was 0.200. The results using other contamination models (mean or variance contamination) are similar (**S5 Fig**). In terms of bias, models were generally least biased when considering inconsistent outliers in the test data.

In general, in the presence of substantial contamination, RMSEs were lowest when estimating GFR based on the six most consistent metabolites, whereas RMSEs were substantially influenced by contamination under the univariate and multivariate outlier methods. These findings remained consistent across each of the contamination models and markers. Similarly, models were least biased when focusing on the most consistent predictor markers. When comparing the robust prediction methods within each outlier strategy, RMSEs was generally best using linear regression and KNN marginalized estimation, though linear regression with screened predictors was a close second, though linear regression with screened consistent markers was the least biased approach.

**Fig 4:**
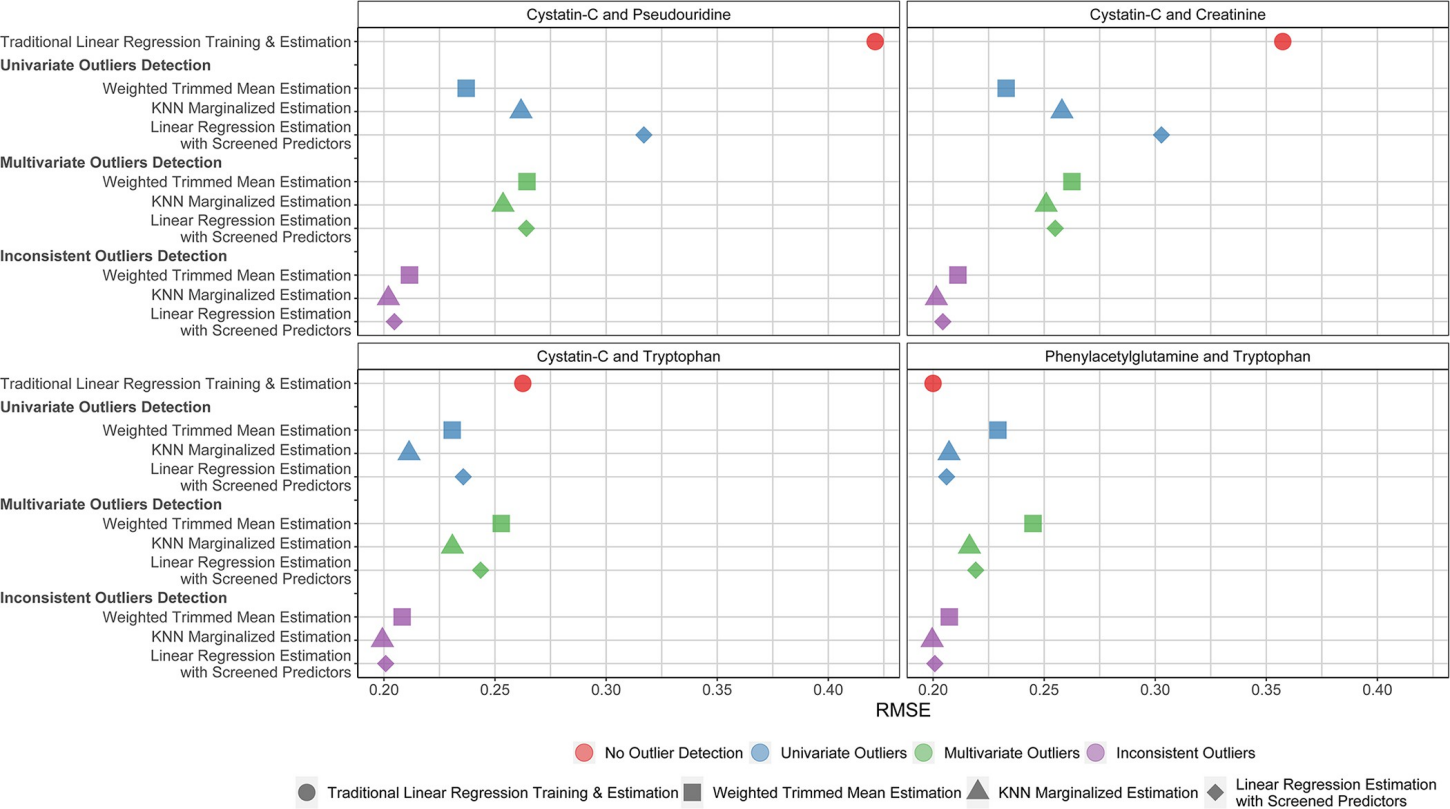
Comparison of RMSE using all outlier detection and robust estimation after mean and variance contamination of two markers. The color of the points represents the underlying outlier detection strategy, and the shape represents the robust estimation approach. Results are averaged across ten cross-validation iterations. We show four of the 28 possible pairs of contaminated markers. The selected pairs represent the results after contaminated two excellent predictors (cystatin-c and pseudouridine, average correlation r with mGFR across studies -0.73 and -0.74, respectively), one excellent predictor and one good predictor (cystatin-c and creatinine, average r for creatinine and mGFR = -0.58), one excellent predictor and one poor predictor (cystatin-c and tryptophan, average r for tryptophan and mGFR = -0.30), and two poor predictors (tryptophan and phenylacetylglutamine, average r for phenylacetylglutamine and mGFR = -0.41). Results are averaged across ten cross-validation iterations. RMSE: Root Mean Square Error.

The RMSEs associated with the contamination of two markers using mean and variance contamination are given in **Fig 4.** Unsurprisingly, contaminating two predictors with high correlation with mGFR (pseudouridine and cystatin-C) of mGFR had a larger impact than contaminating one or no good predictors (RMSE 0.421 using traditional linear regression with no outlier detection). Across all outlier detection methods, again focusing estimation on the consistent markers yielded the smallest RMSE and bias. In terms of estimation, linear regression and KNN marginalized estimation and linear regression with screened predictors were generally similar, though the average bias was slightly smaller using linear regression with screened predictors. For example, after contaminating pseudouridine and cystatin-c, RMSEs ranged between 0.202 and 0.212 when considering the consistent predictors. The RMSE for KNN marginalized estimation and linear regression with screened predictors were similar (e.g., RMSE 0.202 vs 0.205), though the average bias was slightly smaller using linear regression with screened predictors estimation (0.016 vs. 0.011). Similar results are found using mean or variance contamination models (**S6 Fig**).

**Fig 5:**
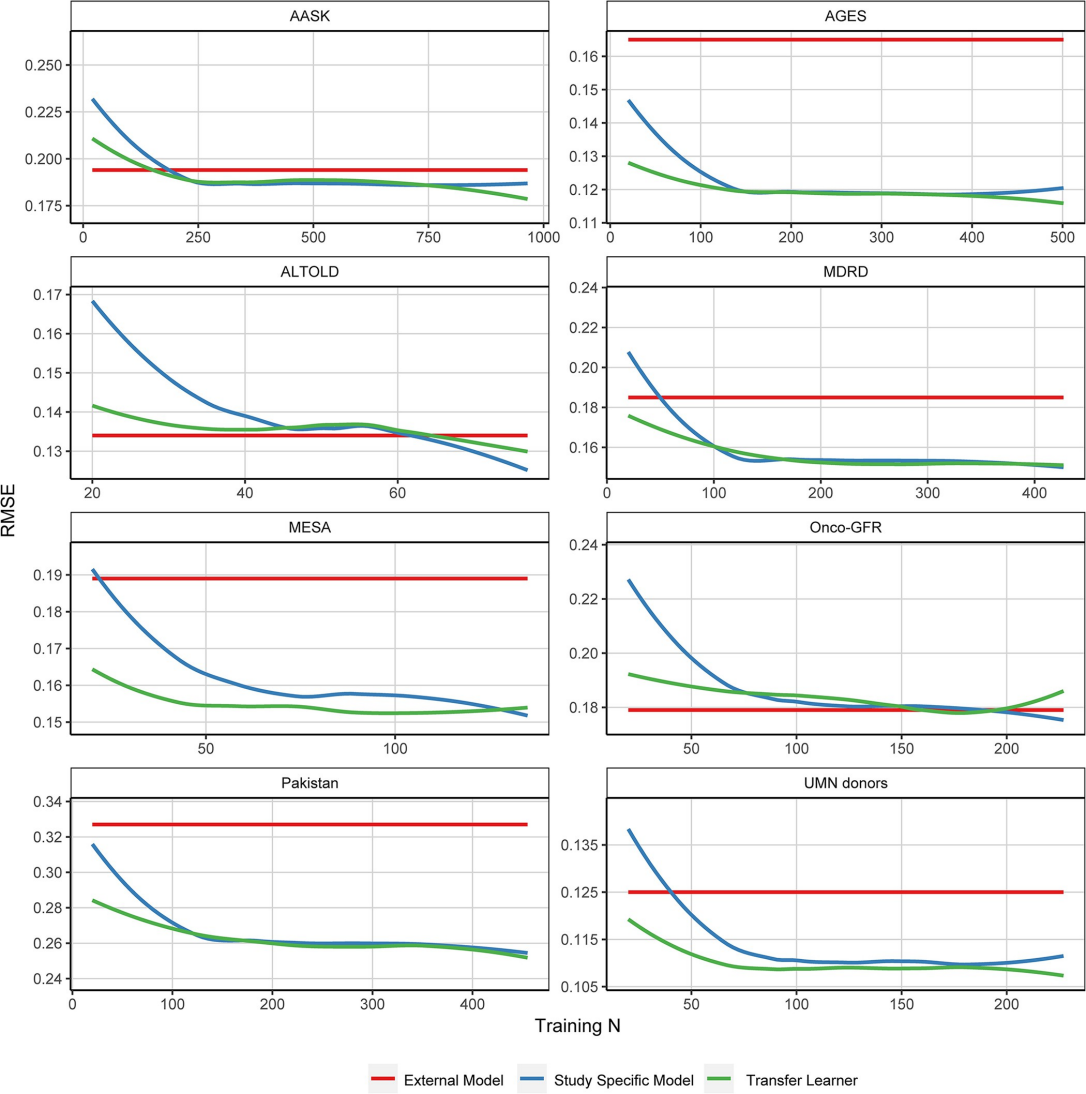
Comparison of RMSEs from naïve (red), study specific linear (blue) and transfer (green) learning models for various training sizes. RMSES are shown on the y-axis and the training sample size is shown in the x-axis. All models include all 8 predictors. RMSEs from linear models that were fit using all studies except for a single held out study used as the test dataset are shown in the horizontal red line (external Model). RMSEs from linear models fit within single studies are shown in blue. In this case, models were developed using a random sample of observations from the given study, and tested on the remaining observations in the study. RMSEs from transfer learning models, shown in green, were developed using a random sample of training observations from the target data and tested on the remaining observations. Given its relatively small total sample size (n = 55), we did not include Crisp in this analysis. Results are averaged across ten cross-validation iterations. RMSE: Root Mean Square Error.

In **Fig 5**, the potential advantages of transfer learning are illustrated. The graph illustrates RMSEs on the y-axis against the training sample size depicted on the x-axis, with all models incorporating 8 predictors. In all panels, the red lines represent the RMSE from a linear regression trained on all studies except the given target application data and then applied to the given target application data (external model). The blue line shows the study-specific linear models trained within the target application data across various randomly selected sample sizes and subsequently tested on the remaining observations. Finally, the green line depicts transfer learning models, developed across a grid of increasing sample sizes and tested on the remaining observations. In some studies, there is an observed improvement in accuracy using transfer learning with relatively small target datasets compared to fitting a new model in the target population. For instance, in both the MESA and Pakistan studies, the transfer learning model exhibited considerably higher accuracy for sample sizes less than 50 in MESA and less than 100 in Pakistan, after which the RMSEs became comparable to linear models. In other studies, such as in Onco-GFR and AASK, a larger target dataset was required to improve estimation beyond the external model. Analogous plots for bias are provided in **S7 Fig.** Both transfer learning and study-specific models exhibited considerably less bias compared to external models models fit to the pooled data set without accounting for study variation.

Lastly, we integrated outlier identification and robust estimation with transfer learning. In general, when using only 25 participants from the target study, combining outlier identification and robust estimation with transfer learning resulted in improved accuracy even further beyond the scenario when no contamination was added (**Table 3**). For example, in MESA the average RMSE from a linear model, developed using a random sample of 25 study participants and applied to the remaining observations in MESA, was 0.180 in the absence of added contamination. However, introducing contamination to one or two predictors in the application data increased the RMSEs to 0.364 and 0.433, respectively. Applying outlier detection and robust estimation using the most consistent markers and linear regression with screened predictors resulted in RMSEs slightly better than when no contamination was added (RMSE under a single contaminated marker and under two contaminated markers 0.173 and 0.173, respectively). The combination of outlier identification and robust prediction with transfer learning yielded further improved RMSEs 0.160 and 0.159, respectively. Bias was generally small after outlier identification (**S4 Table**); the benefit of transfer learning generally was limited to smaller sample sizes. Similarly, when using 50 and 100 observations from the target population, the further application of transfer learning resulted in minimal changes beyond outlier identification in terms of RMSE and bias (**S5–S8 Tables**).

**Table 3:** Summary of RMSEs after combining outlier detection and robust prediction with transfer learning for each study.

Studies	Linear Model, trained on a random sample of 25 observations from given study, tested on remaining study observations.			Linear Model, trained on a random sample of 25 observations from given study, tested on remaining study observations, with outlier detection and robust prediction		Transfer Learning, targeted to random sample of 25 observations from given study, tested on remaining study observations, with outlier identification and robust prediction	
	No added Contamination	Contaminated Single Predictor	Contaminated Two Predictors	Contaminated Single Predictor	Contaminated Two Predictors	Contaminated Single Predictor	Contaminated Two Predictors
**AASK**	0.225	0.430	0.586	0.219	0.222	0.199	0.200
**AGES**	0.151	0.301	0.453	0.141	0.142	0.132	0.133
**ALTOLD**	0.156	0.245	0.429	0.153	0.153	0.143	0.143
**Onco-GFR**	0.221	0.319	0.375	0.203	0.204	0.194	0.194
**MDRD**	0.190	0.364	0.433	0.184	0.185	0.169	0.170
**MESA**	0.180	0.367	0.399	0.173	0.173	0.160	0.159
**Pakistan**	0.333	0.790	0.853	0.304	0.304	0.293	0.293
**UMN DONORS**	0.129	0.274	0.336	0.129	0.129	0.120	0.121

Under no added contamination, we fit linear models developed on a random sample of 25 observations from a given study and applied to the remaining observations from that study. We then added mean and variance contamination to a single excellent predictor (pseudouridine alone) or to two excellent predictors (pseudouridine and cystatin-c) and compared to linear models developed and applied within the given study, linear models developed and applied within the given study but *with outlier identification and robust estimation*, and finally transfer learning models *with outlier identification and robust estimation*. Outliers were identified as the two most inconsistent markers and robust estimation was made using transfer learning models with screen predictors. Results are averaged across ten cross-validation iterations.

RMSE: Root Mean Square Error

## Discussion

Patients with chronic disease or in diverse settings may have inaccurate eGFR because of elevated rates of outlying predictors or systematic differences in non-GFR determinants of one or more filtration markers from the development populations. This paper presents multiple methodologies for outlying predictor identification and robust estimation aimed at improving the accuracy of GFR estimates in patients with chronic diseases. Our findings demonstrate that even in cases of contamination, where a subset of markers are outlying for a particular patient, accurate and unbiased estimates remain feasible. Our results showed that if up to two markers are outlying for a given individual, we can still achieve accurate GFR estimates using the remaining six markers. In the presence of substantial contamination, applying linear regression using the most consistent markers resulted in the most robust estimation in terms of best accuracy and least bias. Further, transfer learning can be a useful tool to tailor equations to specific groups of patients, especially if data is limited in the target population. We found that if only a small dataset is available from a unique population with high rates of outliers, combining outlier identification and robust prediction with transfer learning can address both sources of error. However, once the target data available grows large, the benefit of transfer learning over a model developed directly in the target population becomes minimal.

Clinical practice guidelines recommend the initial evaluation of GFR using creatinine, and subsequently, in cases where eGFRcr proves inaccurate or where highly accurate GFR estimates are required, eGFRcys and eGFRcr-cys are recommended as confirmatory tests [7]. However, when creatinine and cystatin-C based estimates disagree for a given patient, it can be difficult to adjudicate which is more accurate with only two markers. In general, eGFRcr-cys has been shown to be the most accurate in studies where this has been evaluated, but this is not expected to be the case for all individuals [9, 39–43], as individuals with chronic diseases are not likely to be included in these studies. More generally, there are two significant drawbacks to eGFRcr-cys as a confirmatory test [17]. First, eGFRcr-cys is not independent of eGFRcr, thus it does not meet the requirement for a true confirmatory test. Second, with only two markers, any discrepancies between them cannot be easily adjudicated as to which is correct because the non-GFR determinants of cystatin-C are not well understood. For example, in patients with cirrhosis, liver transplant, heart failure, neuromuscular disease, and critical illness, there is variation in the relative performance of eGFRcr vs. eGFRcys, and here eGFRcr-cys was not consistently more accurate [16].

We found the outlier strategey mattered more than the estimation approach. Across all outlier strategies, robust estimation improved on naïve estimates. However, relying solely on the univariate distribution for outlier detection proved ineffective, particularly when employing a mean contamination model (**S8 Fig**). To identify most of the problematic points, most of the distribution of the contaminated markers would need to shift well beyond the 99^th^ percentile (our definition of an upper-tailed outlier), which is especially apparent under a mean contamination model. Consequently, this method is only effective when marker values deviate to an extreme degree, as smaller shifts would remain undetected. Winsorization, despite its merits, poses drawbacks in its treatment of patients with identified problematic values, characterized by a large multivariate Mahalanobis distance from the development data. In this approach, all eight markers undergo uniform shrinkage toward the development data, impacting not only the contaminated markers but also the uncontaminated ones. Consequently, this method may only be useful when all markers exhibit problematic behavior, a scenario in which accurate estimation is unlikely.

Additionally, there are situations in which the transfer learner fit with small sample sizes is slightly worse than the naïve model (for example for smaller sample sizes in Onco-GFR and ALTOLD). In these settings, a small sample may lead to overfitting by not capturing the full variability in the target applications. If a transfer learner is used in these situations, the development data would need to be chosen very carefully to fully represent the target population and minimize negative transfer. Nevertheless, we showed that it is generally possible to accurately estimate GFR given outlying predictors. In practice, the optimal application of GFR estimating equations could apply these methods if multiple markers are measured.

The robust prediction approaches presented here can be implemented using pre-constructed component models so that there is minimal additional computation time compared to a simpler approach such as a single linear model. There are 28 different models comprising six of the eight filtration markers that we evaluated; these models can be pre-fitted in the development data at the outset. In the era of advanced EHR and integrated laboratory information systems within these EHRs, the multiple algorithms required could be embedded into laboratory reporting procedures.

To our knowledge, this work is the first to address the handling of outliers or anomalous predictors in application data. The strengths of our study include our characterization of the two ways in which GFR estimates are altered by the non-GFR determinants of the predictor variables. While the present paper focuses on how to robustly predict GFR in new applications, the methods developed here may be applicable to other clinical algorithms susceptible to outliers.

There are also several areas for improvement in future work. We did not consider the presence of outlying predictors and outliers within the development data. To do so was beyond the scope of this paper, as our focus was solely on anomalies in application data. Future work should investigate the presence and influence of outliers within the development datasets, as there is substantial statistical literature in this area with some reassurance that positive and negative outliers have effects that can cancel each other on model estimates. Further, our method for identifying inconsistent markers could be improved. It is possible that multiple outlying markers could interact with one another; this situation may be more difficult to identify using the methods presented here. Additionally, we were limited to only 8 filtration markers in our analysis. With a larger set of markers, we could employ more sophisticated anomaly detection algorithms like Isolation Forests [44] and Local Outlier Factors (LOF) [45]. These strategies would allow for the possibility that all markers are sufficiently consistent, so we could retain more markers for estimation while still effectively identifying outliers. Additionally, future work could incorporate additional covariates, such as body size, to help identify outlying predictor variables. Lastly, we could explore more advanced models that can be integrated with transfer learning, such as neural networks.

While this analysis focused on the set of markers available to us at this time, our ultimate goal is to apply these methods to a metabolite panel eGFR that is currently under development. For this panel, we have identified 17 candidate markers using untargeted metabolomics data from seven diverse studies. We are presently in the process of developing and validating the targeted mass spectrometry (MS) assay for the panel of markers to assess their analytical precision and to evaluate the performance of a panel eGFR in comparison to mGFR across diverse clinical settings, and the methods developed here may be a useful tool to increase the robustness of panel eGFR in these settings.

Overall, the methods presented provide a specific application for GFR estimation with broader implications. When an underlying process, such as organ function, requires precise estimation beyond what is possible with any single marker due to contamination by biologic processes other than the one being measured, a solution is still possible, using multiple markers.

## Supporting information

S1 File (DOCX)

S2 File (DOCX)

S1 Fig Hypothetical example of data cleaning using bivariate Winsorization. (DOCX)

S2 Fig Example of contamination models using pseudouridine. (DOCX)

S3 Fig Cross-validated RMSEs for all possible subsets of the eight markers. (DOCX)

S4 Fig Comparison of RMSE using all outlier detection and robust prediction approaches with no added contamination. (DOCX)

S5 Fig Comparison of RMSE (A) and Bias (B) using all outlier detection and robust prediction approaches after contamination of each marker individually.
(DOCX)

S6 Fig Comparison of RMSE(A) and Bias (B) using all outlier detection and robust prediction approaches after contamination of two markers.
(DOCX)

S7 Fig Comparison of bias from an external model (red), study specific linear (blue) and transfer (green) learning models for various training sizes. (DOCX)

S8 Fig Illustration of why identifying outlying points based on the extreme quantiles of the univariate distribution of the markers failed to identify many of the contaminated points. (DOCX)

S1 Table Descriptions of study populations. (DOCX)

S2 Table Univariable regression models for mGFR by study. (DOCX)

S3 Table Multivariable regression models for mGFR within study. (DOCX)

S4 Table Summary of bias (log mGFR—log eGFR, log ml/min/1.73 m2) after combining outlier detection and robust prediction with transfer learning using n = 25 for each study. (DOCX)

S5 Table Summary of RMSEs after combining outlier detection and robust prediction with transfer learning using n = 50 for each study. (DOCX)

S6 Table Summary of bias (log mGFR—log eGFR, log ml/min/1.73 m2) after outlier detection and robust prediction with transfer learning using n = 50 for each study. (DOCX)

S7 Table Summary of RMSEs after outlier detection and robust prediction with transfer learning using n = 100 for each study. (DOCX)

S8 Table Summary of bias (log mGFR—log eGFR, log ml/min/1.73 m2) after combining outlier detection and robust prediction with transfer learning using n = 100 for each study. (DOCX)
