# Supplementary material for: Panel estimated Glomerular Filtration Rate (GFR): Statistical considerations for maximizing accuracy in diverse clinical populations
Source: PLoS One. 2024 Dec 2;19(12):e0313154. doi: 10.1371/journal.pone.0313154 (PMC11611103; doi:10.1371/journal.pone.0313154)
Supplement: S1 File — (DOCX) [file pone.0313154.s001.docx]

# **Study Collaborators and Funding**

**Study Collaborators**

**The Age, Gene/Environment Susceptibility Reykjavik study (AGES-RS):** Margret B Andresdottir, Hrefna Gudmundsdottir, Olafur S Indridason and Runolfur Palsson**; Assessing Long Term Outcomes in Living Kidney Donors (ALTOLD):** Bertram Kasiske, Matthew Weir, Todd Pesavento, Roberto Kalil; **Consortium for Radiologic Imaging Studies of Polycystic Kidney Disease (CRISP):** Arlene B Chapman, Douglas P. Landsittel, Michal Mrug, Alan SL Yu**; Multi Ethnic Study of Atherosclerosis (MESA):** Tariq Shafi, Wendy Post, Peter Rossing**; Onco-GFR Study:** Emmanuel de Almeida Burdmann, Renato Antunes Caires**; Pakistan Study:** Saleem Jessani, Rasool Bux, Zainab Samad, Nish Chaturvedi.

**Funding**

**AASK:** Support from NIDDK U01 DK045388 and the NCMHHD M01 RR00071

**AGES:** The AGES-Kidney study is supported by grants from the National Institute of Diabetes and Digestive and Kidney Diseases (R01-DK082447 and supplement 01A1S1 to A.S.L.)

**ALTOLD:** study was funded by the National Institutes of Health (NIH) under the cooperative agreement U01 DK066013. The NIH participated in the interpretation of data, writing the report, and the decision to submit the report for publication. This study was also supported by the Minneapolis Medical Research Foundation, Minneapolis, MN, which did not participate in any aspect of the study.

**CRISP:** The CRISP study is supported by cooperative agreements from the National Institute of Diabetes and Digestive and Kidney Diseases (NIDDK) of the National Institutes of Health (DK056943, DK056956, DK056957, DK056961), and by R01 DK113111. This study was also supported in part by the NIDDK through P30 grants to the Kansas PKD Research and Translation Core Center (DK106912) and the Mayo Translational PKD Center (DK090728), by the National Center for Research Resources General Clinical Research Centers at each institution (RR000039, Emory University; RR00585, Mayo College of Medicine; RR23940, Kansas University Medical Center; RR000032, University of Alabama at Birmingham), and the National Center for Advancing Translational Sciences Clinical and Translational Science Awards at each institution (RR025008 and TR000454, Emory; RR024150 and TR000135, Mayo College of Medicine; RR033179 and TR000001, Kansas University Medical Center; RR025777, TR000165 and TR001417, University of Alabama at Birmingham; RR024153 and TR000005, University of Pittsburgh School of Medicine). The investigators are indebted to the study coordinators in CRISP.

**MDRD:** Support from NIDDK U01 DK35073

**MESA:** This research was supported by contracts 75N92020D00001, HHSN268201500003I, N01-HC-95159, 75N92020D00005, N01-HC-95160, 75N92020D00002, N01-HC-95161, 75N92020D00003, N01-HC-95162, 75N92020D00006, N01-HC-95163, 75N92020D00004, N01-HC-95164, 75N92020D00007, N01-HC-95165, N01-HC-95166, N01-HC-95167, N01-HC-95168 and N01-HC-95169 from the National Heart, Lung, and Blood Institute, and by grants UL1-TR-000040, UL1-TR-001079, and UL1-TR-001420 from the National Center for Advancing Translational Sciences (NCATS). The authors thank the other investigators, the staff, and the participants of the MESA study for their valuable contributions. A full list of participating MESA investigators and institutions can be found at http://www.mesa-nhlbi.org.

**UMN Donors:** no funding to report

**Pakistan Study:** This study was funded by the NIH-Fogarty International Center
