# Supplementary material for: Panel estimated Glomerular Filtration Rate (GFR): Statistical considerations for maximizing accuracy in diverse clinical populations
Source: PLoS One. 2024 Dec 2;19(12):e0313154. doi: 10.1371/journal.pone.0313154 (PMC11611103; doi:10.1371/journal.pone.0313154)
Supplement: S2 File — (DOCX) [file pone.0313154.s002.docx]

# **Supplementary Methods**

*Simulation of application data*

Let $X$ be the $n$ by $8$ matrix of the predictor markers that is randomly split into $X_{train}$ and $X_{test}$. We first similated $X$ as multivariate normal with mean vector $\mu=(\mu_{1}, ...,\mu_{8})$and variance-covariance matrix $\Sigma=\left[ \begin{matrix} \sigma_{11} & \cdots& \sigma_{18} \\ \vdots& \ddots& \vdots\\ \sigma_{81} & \cdots& \sigma_{88} \end{matrix} \right]$. Next, we contaminated a single one marker at a time, leaving the remaining seven markers as observed. Without loss of generality, consider first contaminating$X_{1}$. The conditional distribution of $X_{1}$, given the remaining markers $X_{2}=x_{2},$…, $X_{8}=x_{8}$, is univariate normal^39^ with

mean $\mu_{1}+(\sigma_{12}, ..., \sigma_{18})\left[ \begin{matrix} \sigma_{22} & \cdots& \sigma_{28} \\ \vdots& \ddots& \vdots\\ \sigma_{82} & \cdots& \sigma_{88} \end{matrix} \right]^{-1}[(x_{2}, ..., x_{8})-(\mu_{2}, ...,\mu_{8})]$and

variance $\sigma_{11}$ - $(\sigma_{12}, ..., \sigma_{18}) \left[ \begin{matrix} \sigma_{22} & \cdots& \sigma_{28} \\ \vdots& \ddots& \vdots\\ \sigma_{82} & \cdots& \sigma_{88} \end{matrix} \right]^{-1}(\sigma_{12}, ..., \sigma_{18})'$. Then, to contaminate the distribution of $X_{1}|X_{2}=x_{2},$… $X_{8}=x_{8}$, we altered the mean defined above by adding $\log a$ (which corresponds to multipying the mean in its non-logged scale by $a$) and increased the standard deviation by adding $\log b$. Contaminating the mean may represent scenarios when certain patients consistently have unusually high or low values of certain markers, such as patients with anorexia who tend to have low levels of creatinine. Contaminating the variance may represent scenarios when patients have increased variation in their marker values, for example, the initiation or cessation of medications and treatments. For subjects in $X_{test}$ (corresponding to matrix rows) selected to have outlier predictors, we replaced their values of $X_{1}$with a draw from the contaminated distribution of $X_{1}|X_{2}=x_{2},$… $X_{8}=x_{8}$. The same process was repeated for constructing analogous testing datasets with $X_{2}$ contaminated, $X_{3}$ contaminated, and so on.

We followed a similar procedure to contaminate two markers at time. Here, consider contaminating $X_{1}$and $X_{2}$. The joint distribution of $X_{1}$and$X_{2}$ given the remaining markers $X_{3}=x_{3},$…, $X_{8}=x_{8}$ is bivariate normal with

mean $\left[ \begin{matrix} \mu_{1} \\ \mu_{2} \end{matrix} \right]+\left[ \begin{matrix} \sigma_{13} & ... & \sigma_{18} \\ \sigma_{23} & ... & \sigma_{28} \end{matrix} \right]\left[ \begin{matrix} \sigma_{33} & \cdots& \sigma_{38} \\ \vdots& \ddots& \vdots\\ \sigma_{83} & \cdots& \sigma_{88} \end{matrix} \right]^{-1}[(x_{3}, ..., x_{8})-(\mu_{3}, ...,\mu_{8})]$ and

covariance $\left[ \begin{matrix} \sigma_{11} & \sigma_{12} \\ \sigma_{21} & \sigma_{22} \end{matrix} \right]$ - $\left[ \begin{matrix} \sigma_{13} & ... & \sigma_{18} \\ \sigma_{23} & ... & \sigma_{28} \end{matrix} \right] \left[ \begin{matrix} \sigma_{33} & \cdots& \sigma_{38} \\ \vdots& \ddots& \vdots\\ \sigma_{83} & \cdots& \sigma_{88} \end{matrix} \right]^{-1}\left[ \begin{matrix} \sigma_{13} & ... & \sigma_{18} \\ \sigma_{23} & ... & \sigma_{28} \end{matrix} \right]^{'}$. Again, we contaminated this distribution by adding $\log a$ to the mean elements and $\log b$ to the standard deviations in the bivariate distribution. Note that we increase the standard deviations while keeping the correlation matrix $R$between the markers from the conditional distribution the same (i.e., the contaminated variance-covariance matrix is $\left[ \begin{matrix} \sigma_{11|X_{3}=x_{3},\ldots, X_{8}=x_{8}}+\log b & 0 \\ 0 & \sigma_{22|X_{3}=x_{3},\ldots, X_{8}=x_{8}}+\log b \end{matrix} \right]R\left[ \begin{matrix} \sigma_{11|X_{3}=x_{3},\ldots, X_{8}=x_{8}}+\log b & 0 \\ 0 & \sigma_{22|X_{3}=x_{3},\ldots, X_{8}=x_{8}}+\log b \end{matrix} \right]$.

We only contaminated observations $X_{test}$, and do not contaminate the mGFR outcomes in $y_{test}$. We compared three different types of contamination: mean contamination (letting $a=5$and $b=1$), variance contamination (letting $a=1$and $b=5$) and both mean and variance contamination (letting $a=5$and $b=5$).
