# Supplementary material for: Panel estimated Glomerular Filtration Rate (GFR): Statistical considerations for maximizing accuracy in diverse clinical populations
Source: PLoS One. 2024 Dec 2;19(12):e0313154. doi: 10.1371/journal.pone.0313154 (PMC11611103; doi:10.1371/journal.pone.0313154)

# **S1 Fig.** Hypothetical example of data cleaning using bivariate Winsorization.

Contaminated blue points are shrunken towards the development data (red points); the green points show the Winsorized values.


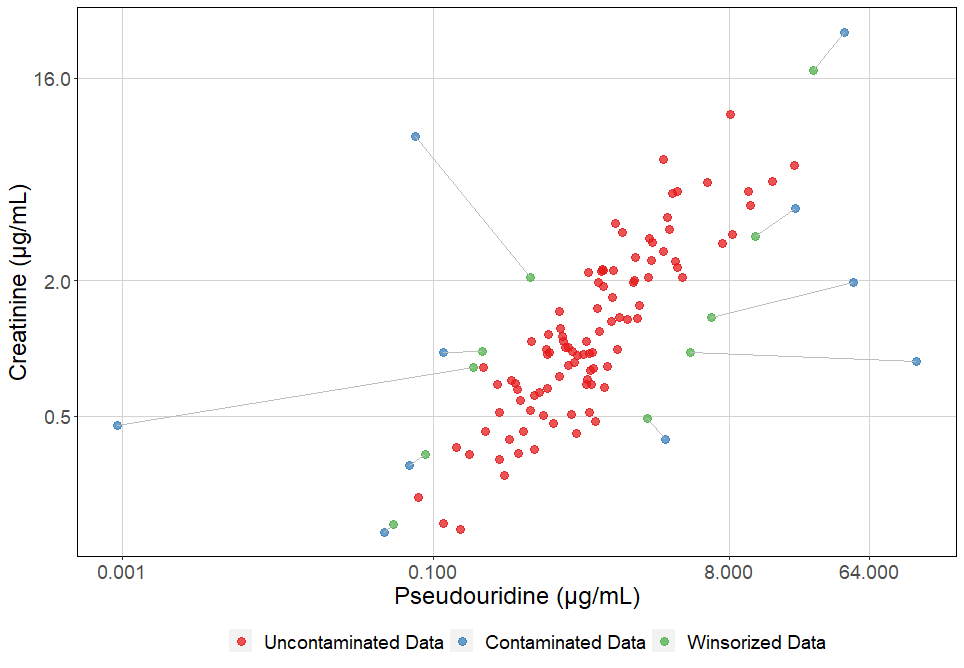

Supplement: S1 Fig — (DOCX) [file pone.0313154.s003.docx]
