# Supplementary material for: Panel estimated Glomerular Filtration Rate (GFR): Statistical considerations for maximizing accuracy in diverse clinical populations
Source: PLoS One. 2024 Dec 2;19(12):e0313154. doi: 10.1371/journal.pone.0313154 (PMC11611103; doi:10.1371/journal.pone.0313154)
Supplement: S2 Fig — (DOCX) [file pone.0313154.s004.docx]

# **S2 Fig.** Example of contamination models using pseudouridine.

A random 10% of pseudouridine values were contaminated.

**
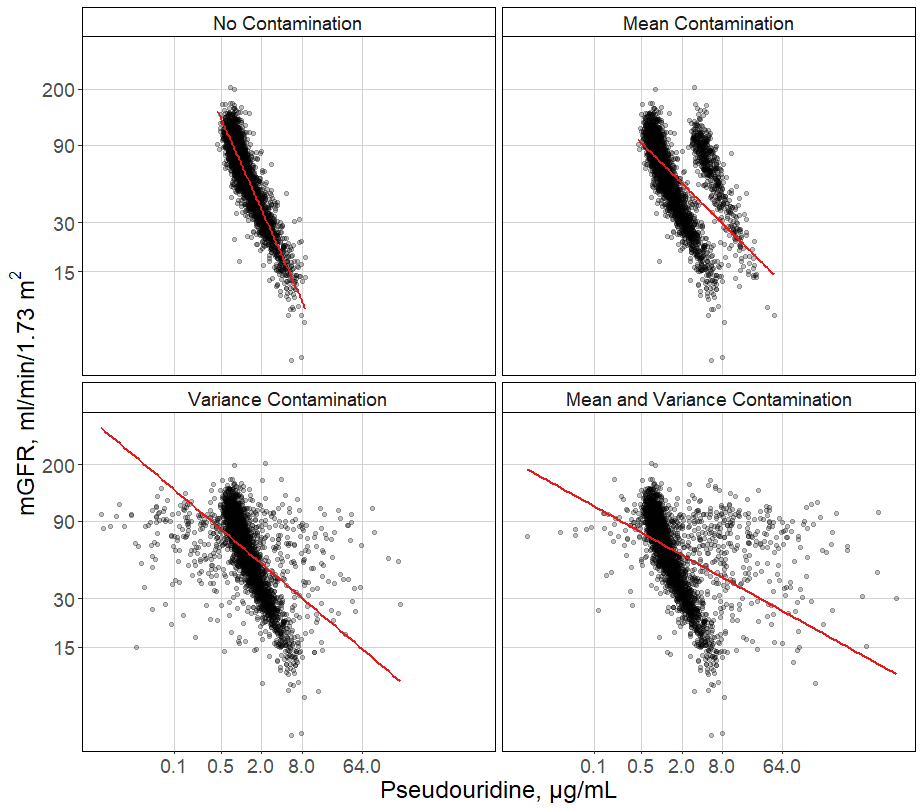
**
