# Supplementary material for: Panel estimated Glomerular Filtration Rate (GFR): Statistical considerations for maximizing accuracy in diverse clinical populations
Source: PLoS One. 2024 Dec 2;19(12):e0313154. doi: 10.1371/journal.pone.0313154 (PMC11611103; doi:10.1371/journal.pone.0313154)

# **S3 Fig.** Cross-validated RMSEs for all possible subsets of the eight markers.

Points represent the cross-validated RMSE for all possible models of the eight markers. The number of possible regression models are provided, the minimum and maximum RMSE are labeled for a given number of markers. Results are averaged across ten cross-validation iterations.

RMSE: Root Mean Square Error


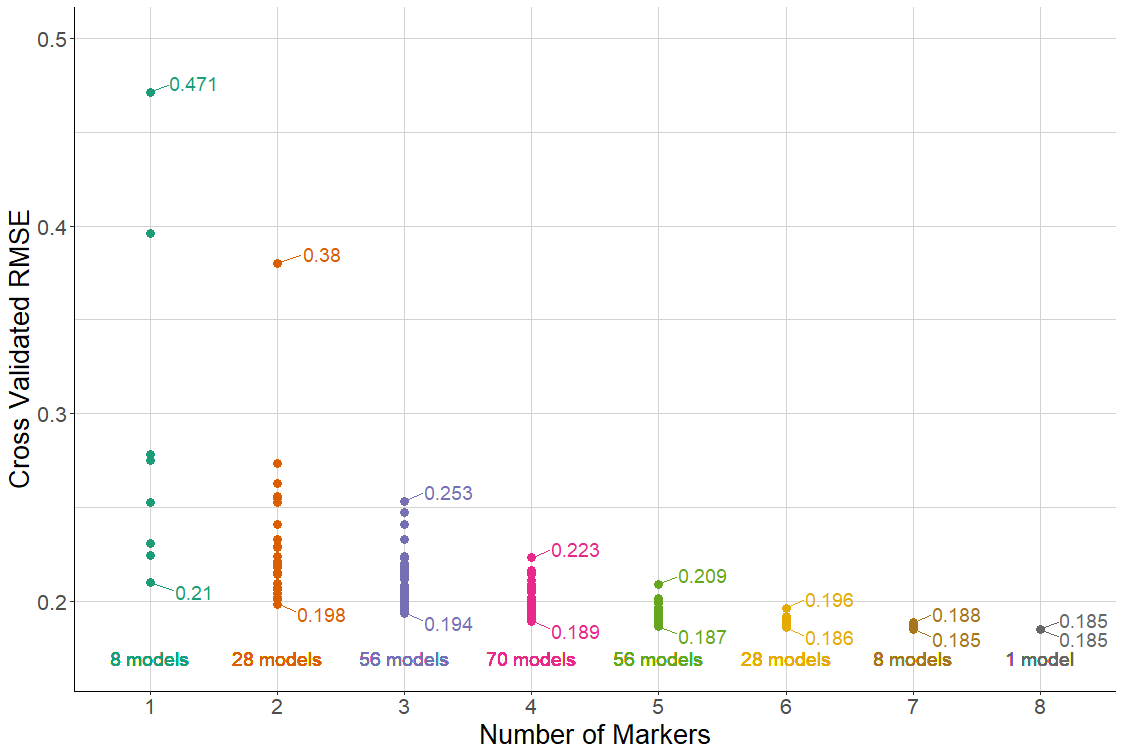

Supplement: S3 Fig — (DOCX) [file pone.0313154.s005.docx]
