# Supplementary material for: Panel estimated Glomerular Filtration Rate (GFR): Statistical considerations for maximizing accuracy in diverse clinical populations
Source: PLoS One. 2024 Dec 2;19(12):e0313154. doi: 10.1371/journal.pone.0313154 (PMC11611103; doi:10.1371/journal.pone.0313154)
Supplement: S4 Fig — (DOCX) [file pone.0313154.s006.docx]

# **S4 Fig.** Comparison of RMSE using all outlier detection and robust prediction approaches with no added contamination.


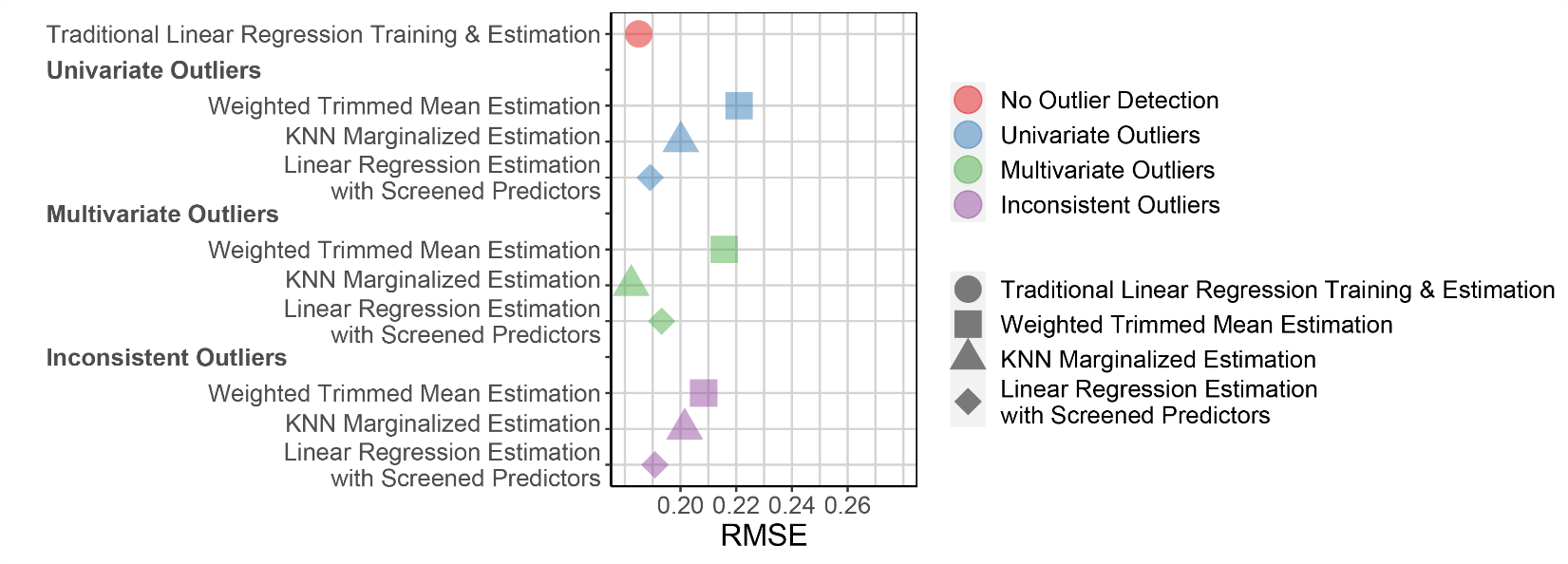


The color of the points represents the underlying outlier detection strategy, and the shape represents the robust estimation approach. Results are averaged across ten cross-validation iterations.
