# Supplementary material for: Panel estimated Glomerular Filtration Rate (GFR): Statistical considerations for maximizing accuracy in diverse clinical populations
Source: PLoS One. 2024 Dec 2;19(12):e0313154. doi: 10.1371/journal.pone.0313154 (PMC11611103; doi:10.1371/journal.pone.0313154)

# **S5 Fig.** Comparison of RMSE (A) and Bias (B) using all outlier detection and robust prediction approaches after contamination of each marker individually.

The color of the points represents the underlying outlier detection strategy, and the shape represents the robust estimation approach. Results are averaged across ten cross-validation iterations. Results are averaged across ten iterations. Bias was calculated as log mGFR –log eGFR and is expressed in log ml/min/1.73m^2^. the dashed line represents a bias of 0. Exponentiated values of bias on the log scale can be interpreted as the ratio of mGFR and eGFR.

RMSE: Root Mean Square Error


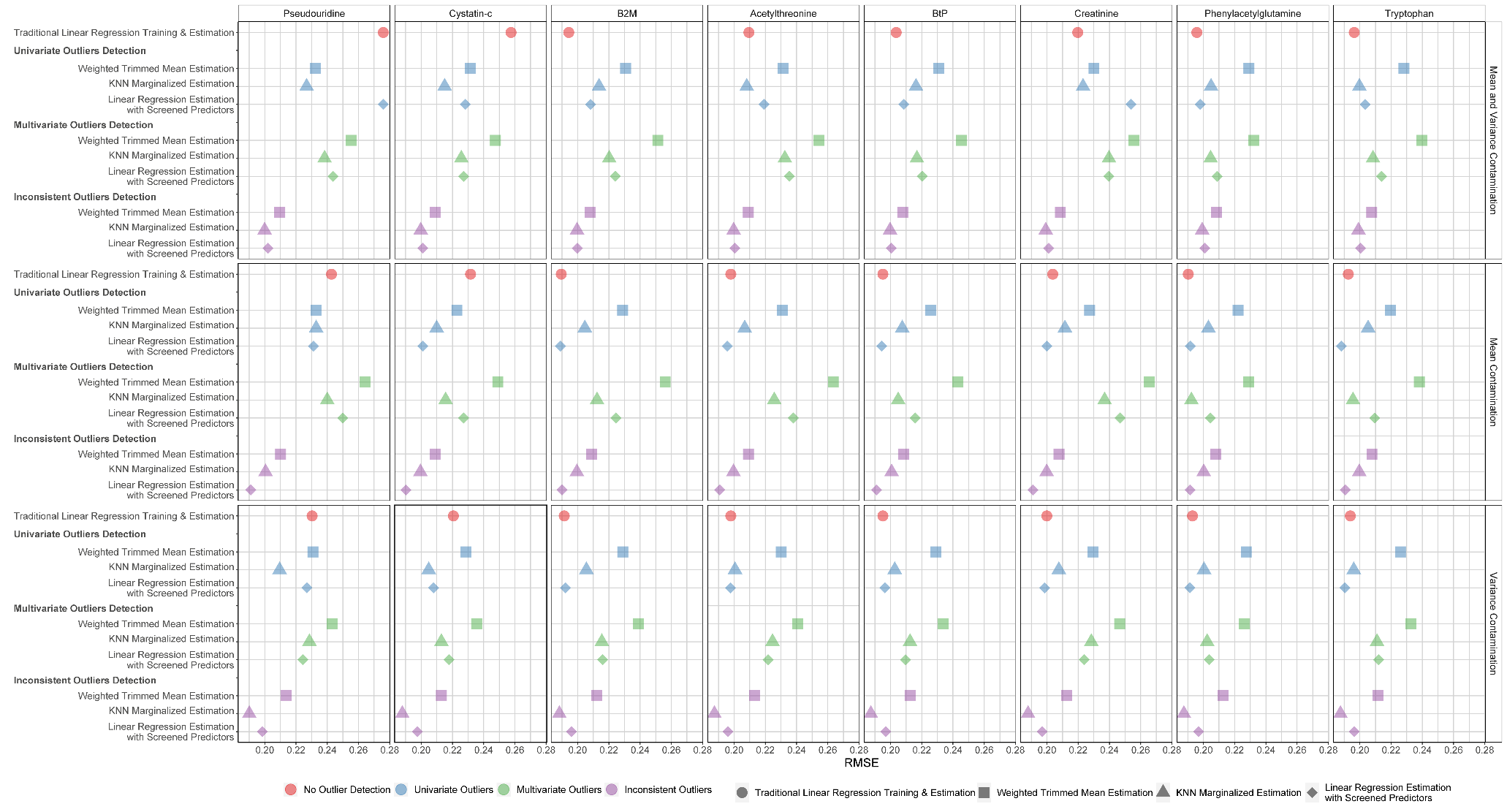


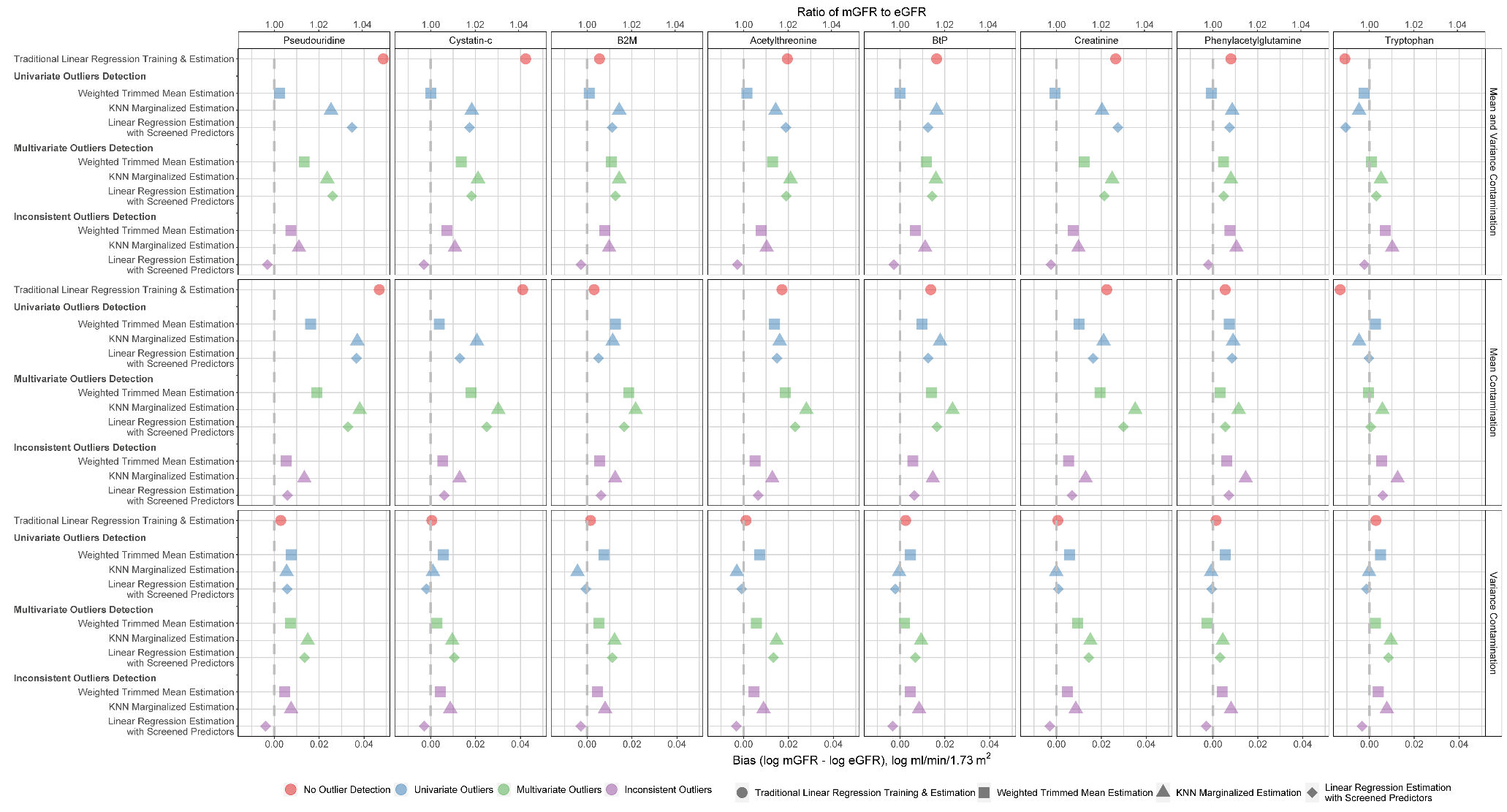

Supplement: S5 Fig — Comparison of RMSE (A) and Bias (B) using all outlier detection and robust prediction approaches after contamination of each marker individually. (DOCX) [file pone.0313154.s007.docx]
