# Supplementary material for: Panel estimated Glomerular Filtration Rate (GFR): Statistical considerations for maximizing accuracy in diverse clinical populations
Source: PLoS One. 2024 Dec 2;19(12):e0313154. doi: 10.1371/journal.pone.0313154 (PMC11611103; doi:10.1371/journal.pone.0313154)

# **S6 Fig.** Comparison of RMSE(A) and Bias (B) using all outlier detection and robust prediction approaches after contamination of two markers.

The color of the points represents the underlying outlier detection strategy, and the shape represents the robust estimation approach. Results are averaged across ten cross-validation iterations. Bias was calculated as log mGFR – log eGFR and is expressed in log ml/min/1.73m^2^. Exponentiated values of bias on the log scale can be interpreted as the ratio of mGFR and eGFR. We show four of the 28 possible pairs of contaminated markers. The selected pairs represent the results after contaminated two excellent predictors (cystatin-C and pseudouridine, average correlation r with mGFR across studies -0.73 and -0.74, respectively), one excellent predictor and one good predictor (cystatin-C and creatinine, average r for creatinine and mGFR = -0.58), one excellent predictor and one poor predictor (cystatin- C and tryptophan, average r for tryptophan and mGFR =-0.30), and two poor predictors (tryptophan and phenylacetylglutamine, average r for phenylacetylglutamine and mGFR =-0.41). Results are averaged across ten cross-validation iterations.

RMSE: Root Mean Square Error

A.


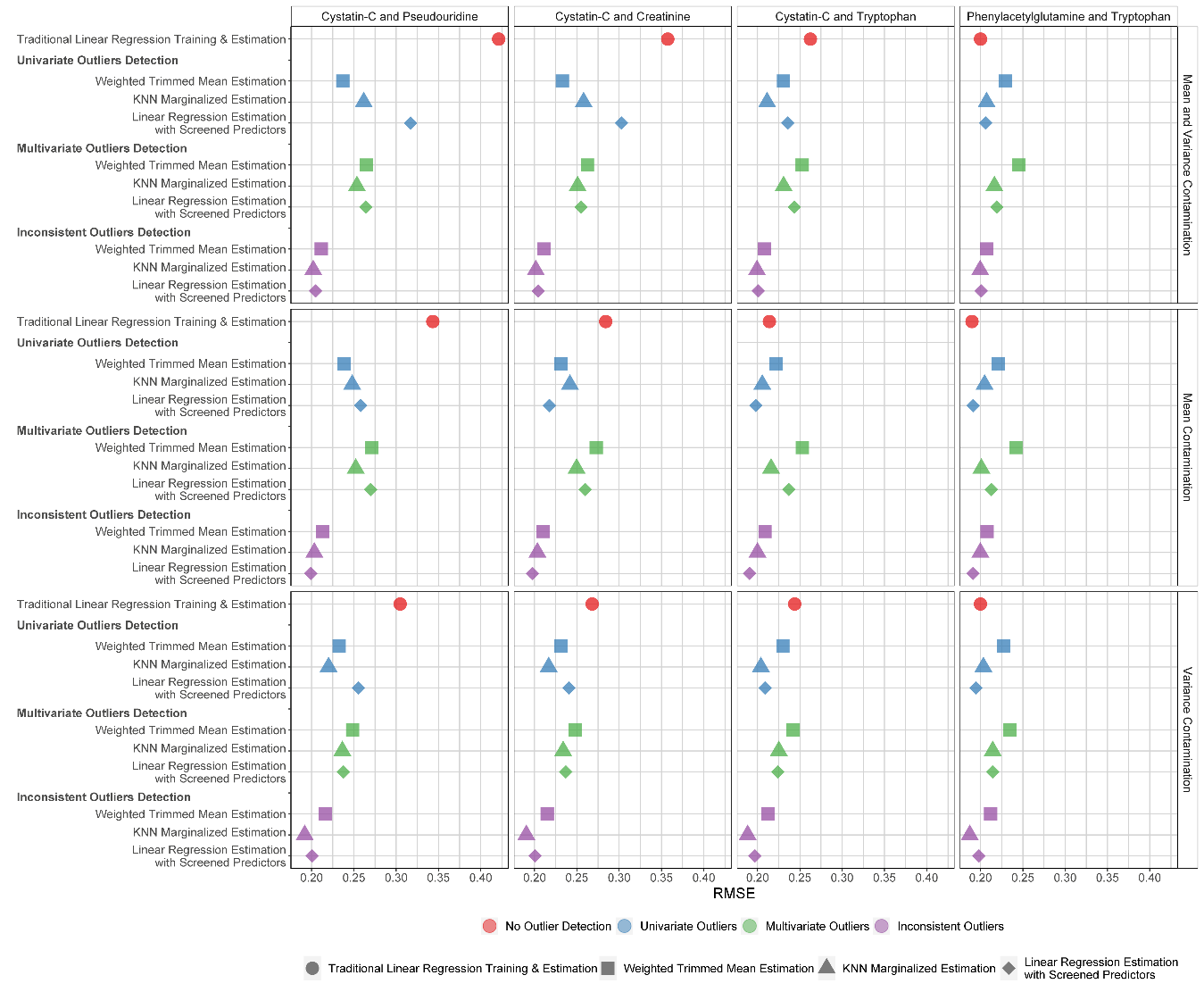


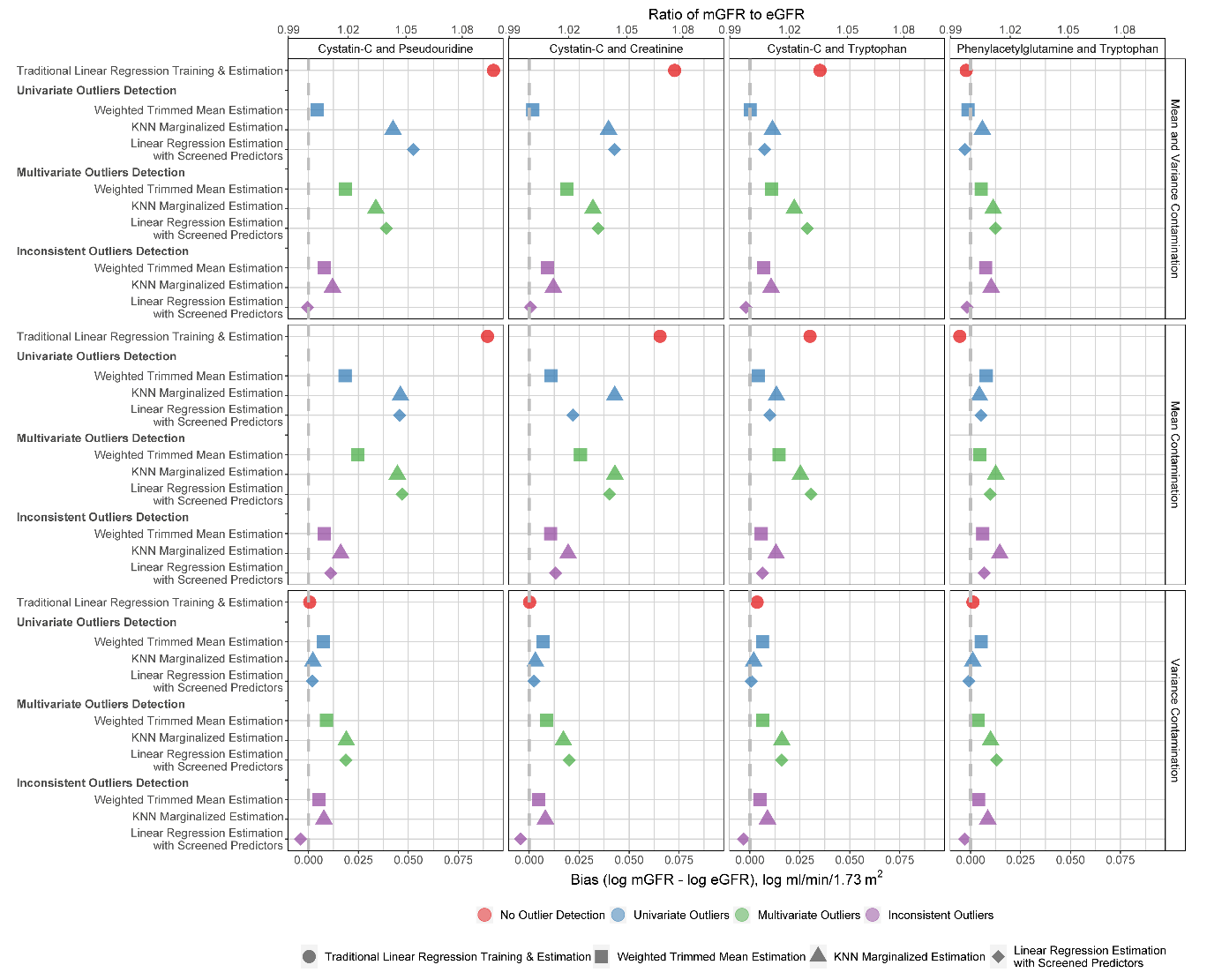

Supplement: S6 Fig — Comparison of RMSE(A) and Bias (B) using all outlier detection and robust prediction approaches after contamination of two markers. (DOCX) [file pone.0313154.s008.docx]
