# Supplementary material for: Panel estimated Glomerular Filtration Rate (GFR): Statistical considerations for maximizing accuracy in diverse clinical populations
Source: PLoS One. 2024 Dec 2;19(12):e0313154. doi: 10.1371/journal.pone.0313154 (PMC11611103; doi:10.1371/journal.pone.0313154)
Supplement: S7 Fig — (DOCX) [file pone.0313154.s009.docx]

**S7 Fig**. Comparison of Bias from an external model (red), study specific linear (blue) and transfer (green) learning models for various training sizes.
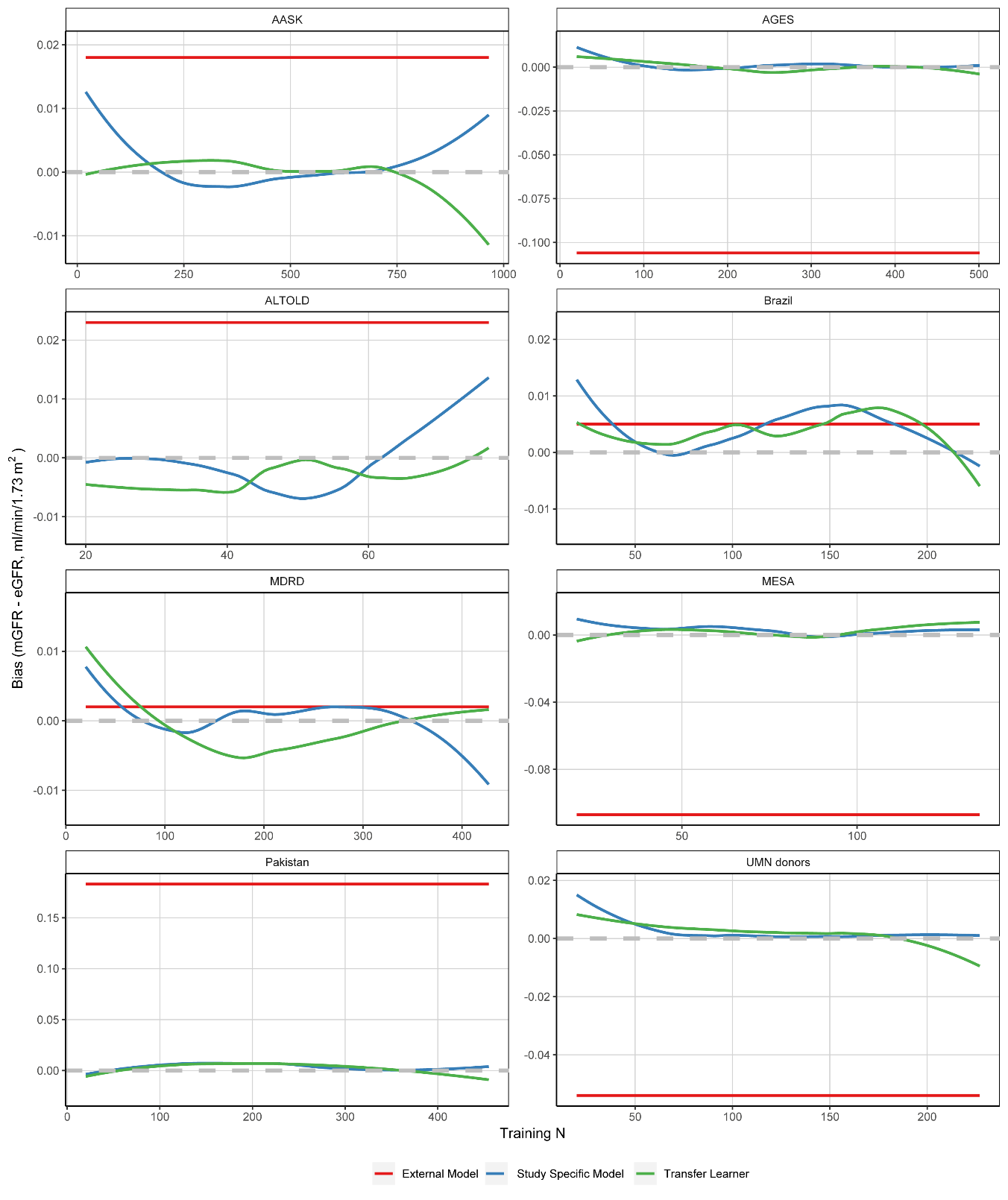


Average bias is shown on the y-axis and the training sample size is shown in the x-axis. All models include all 8 predictors. Average Bias from linear models that were fit using all studies except for a single held out study used as the test dataset are shown in the horizontal red line (External Model). Average bias from linear models fit within single studies are shown in blue. In this case, models were developed using a random sample of observations from the given study and tested on the remaining observations in the study. Average bias from transfer learning models, shown in green, were developed using a random sample of training observations from the target data and tested on the remaining observations. Given its relatively small total sample size (n=55), we did include Crisp in this analysis. Results are averaged across ten cross validation iteration.
