# Supplementary material for: Panel estimated Glomerular Filtration Rate (GFR): Statistical considerations for maximizing accuracy in diverse clinical populations
Source: PLoS One. 2024 Dec 2;19(12):e0313154. doi: 10.1371/journal.pone.0313154 (PMC11611103; doi:10.1371/journal.pone.0313154)

# **S8 Fig.** Illustration of why identifying outlying points based on the extreme quantiles of the univariate distribution of the markers failed to identify many of the contaminated points.

The distribution of pseudouridine in the development data is shown in red, and the 1^st^ and 99^th^ quantile of pseudouridine values are shown in the black dotted lines. The sampled outliers are shown in blue under each model of contamination: mean, variance, or both. Points with values beyond the extreme quantiles would be identified as outliers, while points in between the quantiles would not be identified as outliers.
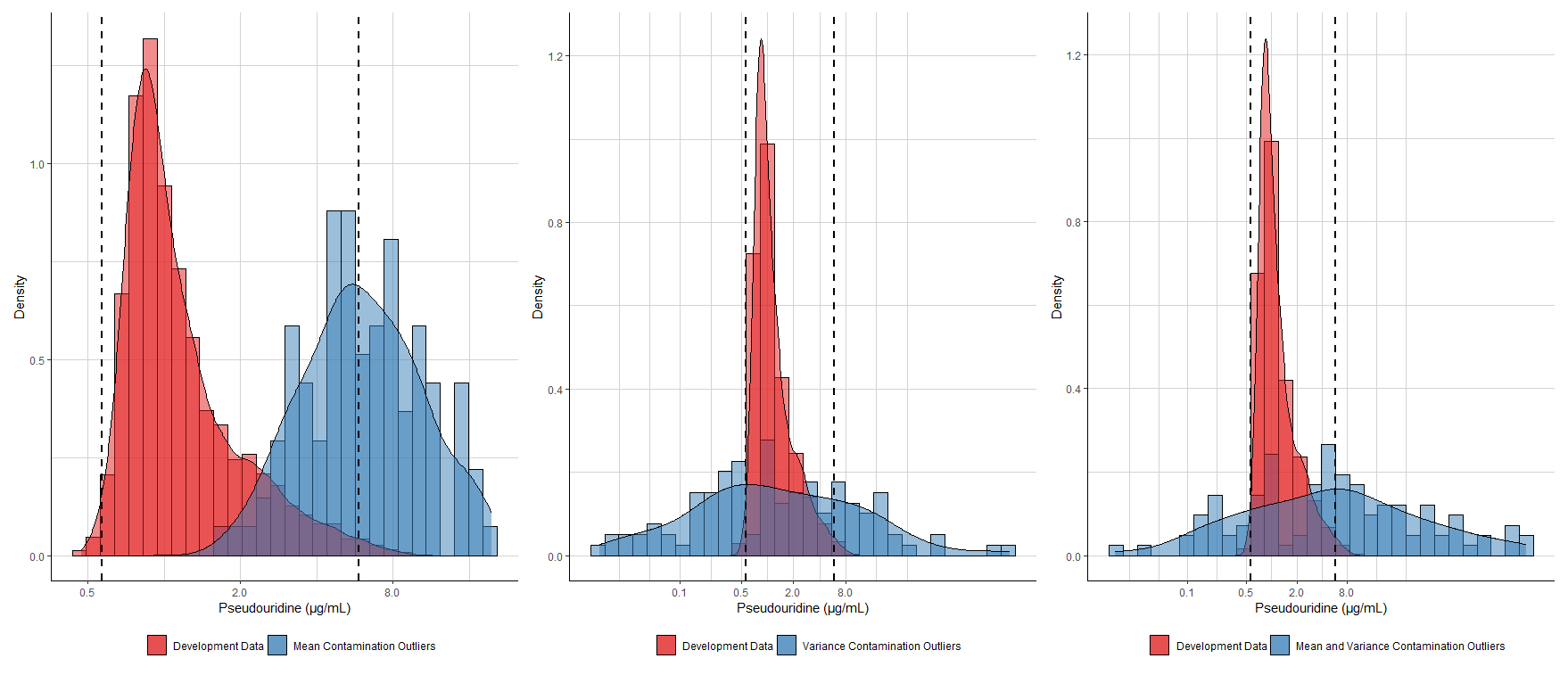

Supplement: S8 Fig — (DOCX) [file pone.0313154.s010.docx]
