# Supplementary material for: Panel estimated Glomerular Filtration Rate (GFR): Statistical considerations for maximizing accuracy in diverse clinical populations
Source: PLoS One. 2024 Dec 2;19(12):e0313154. doi: 10.1371/journal.pone.0313154 (PMC11611103; doi:10.1371/journal.pone.0313154)
Supplement: S1 Table — (DOCX) [file pone.0313154.s011.docx]

# **S1 Table**: Descriptions of study populations

| **Study** | **Study Description** | **mGFR method** |
| --- | --- | --- |
| **AASK^1^** | A multicenter, randomized trial on African Americans aged 18 to 70 years with hypertensive renal disease evaluating the effects of two levels of blood pressure control and medication on GFR | Urinary clearance of iothalamate |
| **AGES^2^** | A longitudinal study in Iceland that investigates the complex interplay between genetics, environmental factors, and aging-related diseases in Iceland | Plasma clearance of iohexol |
| **Altold^3^** | A prospective cohort study of living kidney donors and paired controls | Plasma clearance of iohexol |
| **Onco-GFR^4^** | A prospective cross-sectional study of adult cancer patients in São Paulo, Brazil | Plasma clearance of Cr-EDTA |
| **CRISP^5^** | A cohort study that assesses whether imaging methods can offer precise and consistent indicators of renal disease progression in individuals diagnosed with polycystic kidney disease | Urinary clearance of iothalamate |
| **MDRD^6^** | A multicenter, randomized trial on people with non-diabetic chronic kidney disease that evaluated the effect of dietary protein restriction and strict blood pressure control on the progression of renal disease | Urinary clearance of iothalamate |
| **MESA^7^** | A community-based, multi-ethnic prospective study of adults with subclinical cardiovascular disease. We use the subset of participants recruited into the MESA-Kidney sub-study | Plasma clearance of iohexol |
| **Pakistan^8^** | A Population-based cross-sectional study to evaluate the performance of several GFR estimating equations in a South Asian population based in Pakistan | Urine clearance of Inulin |
| **UMN Donors** | A clinical population of kidney donor candidates at the University of Minnesota. | Plasma clearance of iohexol |

Measured GFR was indexed by body surface area per 1.73 m^2^ using the Dubois formula.

**References**

1. Wright JT, Jr., Bakris G, Greene T, Agodoa LY, Appel LJ, Charleston J, Cheek D, Douglas-Baltimore JG, Gassman J, Glassock R, Hebert L, Jamerson K, Lewis J, Phillips RA, Toto RD, Middleton JP and Rostand SG. Effect of blood pressure lowering and antihypertensive drug class on progression of hypertensive kidney disease: results from the AASK trial. *JAMA*. 2002;288:2421-31.

2. Inker LA, Okparavero A, Tighiouart H, Aspelund T, Andresdottir MB, Eiriksdottir G, Harris T, Launer L, Nikulasdottir H, Sverrisdottir JE, Gudmundsdottir H, Noubary F, Mitchell G, Palsson R, Indridason OS, Gudnason V and Levey AS. Midlife Blood Pressure and Late-Life GFR and Albuminuria: An Elderly General Population Cohort. *Am J Kidney Dis*. 2015;66:240-8.

3. Kasiske BL, Anderson-Haag T, Israni AK, Kalil RS, Kimmel PL, Kraus ES, Kumar R, Posselt AA, Pesavento TE and Rabb H. A prospective controlled study of living kidney donors: three-year follow-up. *American Journal of Kidney Diseases*. 2015;66:114-124.

4. e Silva VTC, Gil Jr LA, Inker LA, Caires RA, Costalonga E, Coura-Filho G, Sapienza MT, Castro Jr G, Estevez-Diz MD and Zanetta DMT. A prospective cross-sectional study estimated glomerular filtration rate from creatinine and cystatin C in adults with solid tumors. *Kidney international*. 2022;101:607-614.

5. Chapman AB, Guay-Woodford LM, Grantham JJ, Torres VE, Bae KT, Baumgarten DA, Kenney PJ, King Jr BF, Glockner JF and Wetzel LH. Renal structure in early autosomal-dominant polycystic kidney disease (ADPKD): The Consortium for Radiologic Imaging Studies of Polycystic Kidney Disease (CRISP) cohort. *Kidney international*. 2003;64:1035-1045.

6. Levey AS, Bosch JP, Lewis JB, Greene T, Rogers N, Roth D and Group* MoDiRDS. A more accurate method to estimate glomerular filtration rate from serum creatinine: a new prediction equation. *Annals of internal medicine*. 1999;130:461-470.

7. Inker LA, Shafi T, Okparavero A, Tighiouart H, Eckfeldt JH, Katz R, Johnson WC, Dermond N, Tariq Z, Benayache I, Post WS, Coresh J, Levey AS and Shlipak MG. Effects of Race and Sex on Measured GFR: The Multi-Ethnic Study of Atherosclerosis. *Am J Kidney Dis*. 2016;68:743-751.

8. Jessani S, Levey AS, Bux R, Inker LA, Islam M, Chaturvedi N, Mariat C, Schmid CH and Jafar TH. Estimation of GFR in South Asians: a study from the general population in Pakistan. *Am J Kidney Dis*. 2014;63:49-58.
