# Supplementary material for: Panel estimated Glomerular Filtration Rate (GFR): Statistical considerations for maximizing accuracy in diverse clinical populations
Source: PLoS One. 2024 Dec 2;19(12):e0313154. doi: 10.1371/journal.pone.0313154 (PMC11611103; doi:10.1371/journal.pone.0313154)
Supplement: S2 Table — (DOCX) [file pone.0313154.s012.docx]

# **S2 Table.** Univariable regression models for mGFR by study.

Each study by marker combination represents a separate model. Standard errors are shown in parentheses.

| **Marker** | **Pseudouridine** | | | | **Cystatin–C** | | | | **B2M** | | | | **Acetylthreonine** | | | | |
| --- | --- | --- | --- | --- | --- | --- | --- | --- | --- | --- | --- | --- | --- | --- | --- | --- | --- |
| **study** | **intercept** | **slope** | **RMSE** | **R^2^** | **intercept** | **slope** | **RMSE** | **R^2^** | **intercept** | **slope** | **RMSE** | **R^2^** | **intercept** | **slope** | **RMSE** | **R^2^** |  |
| AASK | 4.18 (0.01) | -0.92 (0.01) | 0.21 | 0.81 | 4.28 (0.01) | -1.13 (0.02) | 0.23 | 0.76 | 4.86 (0.02) | -0.79 (0.02) | 0.26 | 0.70 | 2.20 (0.03) | -0.88 (0.02) | 0.24 | 0.74 |  |
| AGES | 4.17 (0.01) | -0.83 (0.02) | 0.14 | 0.79 | 4.22 (0.01) | -1.00 (0.02) | 0.14 | 0.78 | 4.90 (0.02) | -0.79 (0.02) | 0.15 | 0.75 | 2.23 (0.05) | -0.85 (0.02) | 0.15 | 0.74 |  |
| ALTOLD | 4.43 (0.03) | -0.40 (0.09) | 0.14 | 0.12 | 4.47 (0.02) | -0.35 (0.08) | 0.14 | 0.14 | 4.80 (0.04) | -0.50 (0.08) | 0.13 | 0.21 | 3.42 (0.24) | -0.44 (0.09) | 0.14 | 0.15 |  |
| Onco-GFR | 4.22 (0.01) | -0.78 (0.04) | 0.21 | 0.55 | 4.38 (0.01) | -0.95 (0.04) | 0.2 | 0.61 | 4.94 (0.04) | -0.79 (0.04) | 0.22 | 0.53 | 2.27 (0.11) | -0.85 (0.04) | 0.21 | 0.56 |  |
| CRISP | 4.29 (0.03) | -0.92 (0.11) | 0.17 | 0.55 | 4.42 (0.03) | -0.95 (0.13) | 0.18 | 0.48 | 4.84 (0.06) | -0.65 (0.09) | 0.18 | 0.52 | 2.04 (0.32) | -0.97 (0.13) | 0.18 | 0.52 |  |
| MDRD | 3.99 (0.02) | -0.67 (0.02) | 0.2 | 0.74 | 4.22 (0.02) | -1.19 (0.03) | 0.19 | 0.76 | 4.77 (0.04) | -0.84 (0.02) | 0.19 | 0.75 | 2.37 (0.03) | -0.7 (0.02) | 0.21 | 0.71 |  |
| MESA | 4.20 (0.02) | -0.72 (0.06) | 0.16 | 0.40 | 4.25 (0.01) | -0.72 (0.07) | 0.17 | 0.36 | 4.72 (0.04) | -0.60 (0.06) | 0.16 | 0.37 | 2.32 (0.19) | -0.78 (0.07) | 0.16 | 0.37 |  |
| Pakistan | 4.37 (0.01) | -1.09 (0.02) | 0.26 | 0.81 | 4.49 (0.01) | -1.27 (0.03) | 0.3 | 0.75 | 5.46 (0.03) | -1.07 (0.03) | 0.3 | 0.75 | 1.80 (0.06) | -1.09 (0.03) | 0.28 | 0.78 |  |
| UMN donors | 4.36 (0.01) | -0.65 (0.05) | 0.12 | 0.40 | 4.36 (0.01) | -0.65 (0.05) | 0.12 | 0.38 | 4.72 (0.02) | -0.45 (0.04) | 0.13 | 0.27 | 3.10 (0.14) | -0.57 (0.05) | 0.13 | 0.28 |  |
| **Marker** | **BTP** | | | | **Serum Creatinine** | | | | **Phenylacetylglutamine** | | | | **Tryptophan** | | | | |
| **study** | **intercept** | **slope** | **RMSE** | **R^2^** | **intercept** | **slope** | **RMSE** | **R^2^** | **intercept** | **slope** | **RMSE** | **R^2^** | **intercept** | **slope** | **RMSE** | **R^2^** |  |
| AASK | 3.86 (0.01) | -0.62 (0.02) | 0.3 | 0.59 | 4.38 (0.01) | -0.93 (0.02) | 0.27 | 0.67 | 3.95 (0.01) | -0.34 (0.01) | 0.38 | 0.35 | 1.71 (0.12) | 0.93 (0.05) | 0.4 | 0.27 |  |
| AGES | 3.99 (0.01) | -0.75 (0.02) | 0.17 | 0.69 | 4.05 (0.01) | -0.77 (0.03) | 0.19 | 0.60 | 4.16 (0.01) | -0.28 (0.02) | 0.26 | 0.25 | 2.69 (0.18) | 0.53 (0.07) | 0.29 | 0.09 |  |
| ALTOLD | 4.45 (0.05) | -0.19 (0.08) | 0.15 | 0.04 | 4.51 (0.02) | -0.16 (0.07) | 0.15 | 0.04 | 4.52 (0.02) | -0.04 (0.02) | 0.15 | 0.02 | 3.97 (0.18) | 0.24 (0.07) | 0.15 | 0.08 |  |
| Onco-GFR | 4.03 (0.02) | -0.77 (0.04) | 0.21 | 0.57 | 4.20 (0.02) | -0.78 (0.05) | 0.23 | 0.51 | 4.24 (0.02) | -0.17 (0.02) | 0.29 | 0.18 | 3.09 (0.20) | 0.49 (0.08) | 0.3 | 0.12 |  |
| Crisp | 4.17 (0.05) | -0.49 (0.08) | 0.2 | 0.39 | 4.42 (0.03) | -0.63 (0.13) | 0.21 | 0.31 | 4.34 (0.05) | -0.16 (0.06) | 0.24 | 0.13 | 3.92 (0.41) | 0.21 (0.16) | 0.25 | 0.03 |  |
| MDRD | 3.87 (0.02) | -0.81 (0.03) | 0.23 | 0.63 | 4.09 (0.02) | -0.85 (0.03) | 0.21 | 0.69 | 3.56 (0.02) | -0.24 (0.02) | 0.32 | 0.31 | 1.66 (0.12) | 0.81 (0.06) | 0.32 | 0.30 |  |
| MESA | 4.13 (0.03) | -0.35 (0.05) | 0.18 | 0.21 | 4.30 (0.02) | -0.24 (0.07) | 0.20 | 0.06 | 4.32 (0.02) | -0.07 (0.02) | 0.2 | 0.05 | 3.94 (0.14) | 0.19 (0.07) | 0.2 | 0.04 |  |
| Pakistan | 4.12 (0.02) | -0.74 (0.03) | 0.38 | 0.59 | 4.18 (0.02) | -0.98 (0.03) | 0.35 | 0.64 | 4.17 (0.02) | -0.36 (0.02) | 0.48 | 0.34 | 2.83 (0.22) | 0.67 (0.10) | 0.56 | 0.08 |  |
| UMN donors | 4.37 (0.03) | -0.20 (0.04) | 0.14 | 0.09 | 4.47 (0.01) | -0.23 (0.05) | 0.14 | 0.08 | 4.47 (0.01) | -0.07 (0.01) | 0.14 | 0.08 | 4.55 (0.15) | -0.01 (0.05) | 0.15 | 0.00 |  |

RMSE: Root Mean Square Error
