# Supplementary material for: Panel estimated Glomerular Filtration Rate (GFR): Statistical considerations for maximizing accuracy in diverse clinical populations
Source: PLoS One. 2024 Dec 2;19(12):e0313154. doi: 10.1371/journal.pone.0313154 (PMC11611103; doi:10.1371/journal.pone.0313154)
Supplement: S3 Table — (DOCX) [file pone.0313154.s013.docx]

# **S3 Table.** Multivariable regression models for mGFR within study.

|  | **AASK** | **AGES** | **ALTOLD** | **Onco-GFR** | **Crisp** | **MDRD** | **MESA** | **Pakistan** | **UMN donors** |
| --- | --- | --- | --- | --- | --- | --- | --- | --- | --- |
| **Intercept** | 3.72 (0.13) | 3.5  (0.15) | 3.63 (0.31) | 3.49 (0.23) | 3.67 (0.64) | 3.77 (0.17) | 2.93 (0.31) | 3.71 (0.30) | 4.03 (0.18) |
| **Coefficients** |  |  |  |  |  |  |  |  |  |
| Pseudouridine | -0.51 (0.05) | -0.29 (0.05) | -0.06 (0.11) | -0.05 (0.08) | -0.28 (0.28) | -0.08 (0.06) | -0.22 (0.12) | -0.71 (0.11) | -0.29 (0.07) |
| Cystatin-C | -0.27 (0.04) | -0.31 (0.05) | -0.18 (0.08) | -0.30 (0.11) | -0.06 (0.25) | -0.37 (0.07) | -0.22 (0.11) | 0.00 (0.12) | -0.30 (0.07) |
| B2M | 0.01 (0.03) | -0.10 (0.04) | -0.29 (0.11) | 0.04 (0.09) | -0.14 (0.02) | -0.21 (0.04) | -0.08 (0.10) | -0.06 (0.09) | -0.07 (0.05) |
| Acetylthreonine | 0.02 (0.04) | -0.06 (0.05) | -0.15 (0.11) | -0.12 (0.07) | -0.17 (0.23) | -0.09 (0.06) | -0.40 (0.11) | -0.22 (0.10) | -0.15 (0.06) |
| BTP | -0.06 (0.02) | -0.14 (0.03) | 0.05 (0.07) | -0.28 (0.06) | -0.17 (0.09) | -0.01 (0.04) | -0.05 (0.05) | -0.04 (0.04) | 0.00 (0.03) |
| Serum Creatinine | -0.13 (0.03) | -0.03 (0.03) | -0.12 (0.08) | -0.24 (0.06) | -0.14 (0.15) | -0.20 (0.04) | 0.10 (0.06) | -0.03 (0.06) | 0.03 (0.04) |
| Phenylacetylglutamine | -0.03 (0.01) | 0.00 (0.01) | -0.02 (0.02) | -0.04 (0.01) | -0.03 (0.04) | -0.01 (0.01) | -0.01 (0.02) | -0.03 (0.02) | -0.04 (0.01) |
| Tryptophan | 0.21 (0.03) | 0.23 (0.03) | 0.25 (0.07) | 0.15 (0.05) | 0.10 (0.12) | 0.16 (0.03) | 0.17 (0.05) | 0.07 (0.06) | 0.00 (0.04) |
| **RMSE** | **0.18** | **0.12** | **0.12** | **0.17** | **0.16** | **0.15** | **0.15** | **0.25** | **0.11** |
| **R^2^** | **0.85** | **0.86** | **0.37** | **0.72** | **0.65** | **0.85** | **0.52** | **0.81** | **0.50** |

RMSE: Root Mean Square Error
