# Supplementary material for: Panel estimated Glomerular Filtration Rate (GFR): Statistical considerations for maximizing accuracy in diverse clinical populations
Source: PLoS One. 2024 Dec 2;19(12):e0313154. doi: 10.1371/journal.pone.0313154 (PMC11611103; doi:10.1371/journal.pone.0313154)
Supplement: S5 Table — (DOCX) [file pone.0313154.s015.docx]

# **S5 Table.** Summary of RMSEs after combining outlier detection and robust prediction with transfer learning using n=50 for each study.

|  | **Linear Model, trained on a random sample of 50 observations from given study, tested on remaining study observations.** | **Linear Model, trained on a random sample of 50 observations from given study, tested on remaining study observations** | | **Linear Model, trained on a random sample of 50 observations from given study, tested on remaining study observations, with outlier identification and robust prediction** | | **Transfer Learning, targeted to random sample of 50 observations from given study, tested on remaining study observations, with outlier identification and robust prediction** | |
| --- | --- | --- | --- | --- | --- | --- | --- |
|  | **No added Contamination** | **Contaminated Single Predictor** | **Contaminated Two Predictors** | **Contaminated Single Predictor** | **Contaminated Two Predictors** | **Contaminated Single Predictor** | **Contaminated Two Predictors** |
| **AASK** | 0.212 | 0.445 | 0.615 | 0.205 | 0.205 | 0.199 | 0.199 |
| **AGES** | 0.127 | 0.242 | 0.313 | 0.128 | 0.128 | 0.124 | 0.124 |
| **ALTOLD** | 0.135 | 0.144 | 0.223 | 0.142 | 0.143 | 0.138 | 0.138 |
| **Onco-GFR** | 0.190 | 0.218 | 0.346 | 0.190 | 0.190 | 0.184 | 0.185 |
| **MDRD** | 0.166 | 0.196 | 0.434 | 0.168 | 0.167 | 0.168 | 0.168 |
| **MESA** | 0.165 | 0.210 | 0.465 | 0.160 | 0.160 | 0.157 | 0.157 |
| **Pakistan** | 0.281 | 0.654 | 0.617 | 0.271 | 0.272 | 0.279 | 0.278 |
| **UMN DONORS** | 0.117 | 0.308 | 0.358 | 0.121 | 0.120 | 0.116 | 0.116 |

RMSE: Root Mean Square Error

Under no added contamination, we fit linear models developed on a random sample of 50 observations from a given study and applied to the remaining observations from that study. We then added mean and variance contamination to a single excellent predictor (pseudouridine alone) or to two excellent predictors (pseudouridine and cystatin-C) and compared to linear models developed and applied within the given study, linear models developed and applied within the given study but *with outlier identification and robust estimation*, and finally transfer learning models *with outlier identification and robust estimation*. Outliers were identified as the two most inconsistent markers and robust prediction was made using transfer learning models with screened predictors.
