# Supplementary material for: Panel estimated Glomerular Filtration Rate (GFR): Statistical considerations for maximizing accuracy in diverse clinical populations
Source: PLoS One. 2024 Dec 2;19(12):e0313154. doi: 10.1371/journal.pone.0313154 (PMC11611103; doi:10.1371/journal.pone.0313154)
Supplement: S6 Table — (DOCX) [file pone.0313154.s016.docx]

**S6 Table.** Summary of Bias (log mGFR - log eGFR, log ml/min/1.73 m^2^) after outlier detection and robust prediction with transfer learning using n=50 for each study.

|  | **Linear Model, trained on a random sample of 50 observations from given study, tested on remaining study observations.** | **Linear Model, trained on a random sample of 50 observations from given study, tested on remaining study observations** | | **Linear Model, trained on a random sample of 50 observations from given study, tested on remaining study observations, with outlier identification and robust prediction** | | **Transfer Learning, targeted to random sample of 50 observations from given study, tested on remaining study observations, with outlier identification and robust prediction** | |
| --- | --- | --- | --- | --- | --- | --- | --- |
|  | **No added Contamination** | **Contaminated Single Predictor** | **Contaminated Two Predictors** | **Contaminated Single Predictor** | **Contaminated Two Predictors** | **Contaminated Single Predictor** | **Contaminated Two Predictors** |
| **AASK** | 0.003 | 0.082 | 0.144 | 0.006 | 0.006 | 0.001 | 0.001 |
| **AGES** | 0.002 | 0.040 | 0.064 | -0.003 | 0.003 | 0.005 | 0.006 |
| **ALTOLD** | -0.001 | -0.004 | 0.032 | -0.003 | -0.003 | 0.001 | -0.001 |
| **Onco-GFR** | 0.0004 | 0.011 | 0.052 | 0.002 | 0.017 | -0.003 | -0.003 |
| **MDRD** | 0.002 | -0.005 | -0.095 | -0.0003 | -0.0004 | 0.003 | 0.004 |
| **MESA** | -0.008 | 0.021 | 0.083 | -0.005 | -0.007 | -0.009 | -0.010 |
| **Pakistan** | 0.001 | 0.122 | 0.104 | 0.005 | 0.004 | 0.005 | 0.005 |
| **UMN DONORS** | 0.0003 | 0.065 | 0.075 | -0.003 | -0.003 | 0.001 | 0.0002 |

Under no added contamination, we fit linear models developed on a random sample of 50 observations from a given study and applied to the remaining observations from that study. We then added mean and variance contamination to a single excellent predictor (pseudouridine alone) or to two excellent predictors (pseudouridine and cystatin-C) and compared to linear models developed and applied within the given study, linear models developed and applied within the given study but *with outlier identification and robust estimation*, and finally transfer learning models *with outlier identification and robust estimation*. Outliers were identified as the two most inconsistent markers and robust prediction was made using transfer learning models with screen predictors. Results are averaged across ten cross-validation iterations.
