# Supplementary material for: Panel estimated Glomerular Filtration Rate (GFR): Statistical considerations for maximizing accuracy in diverse clinical populations
Source: PLoS One. 2024 Dec 2;19(12):e0313154. doi: 10.1371/journal.pone.0313154 (PMC11611103; doi:10.1371/journal.pone.0313154)
Supplement: S7 Table — (DOCX) [file pone.0313154.s017.docx]

# **S7 Table**. Summary of RMSEs after outlier detection and robust prediction with transfer learning using n=100 for each study.

|  | **Linear Model, trained on a random sample of 100 observations from given study, tested on remaining study observations.** | **Linear Model, trained on a random sample of 100 observations from given study, tested on remaining study observations** | | **Linear Model, trained on a random sample of 100 observations from given study, tested on remaining study observations, with outlier identification and robust prediction** | | **Transfer Learning, targeted to random sample of 100 observations from given study, tested on remaining study observations, with outlier identification and robust prediction** | |
| --- | --- | --- | --- | --- | --- | --- | --- |
|  | **No added Contamination** | **Contaminated Single Predictor** | **Contaminated Two Predictors** | **Contaminated Single Predictor** | **Contaminated Two Predictors** | **Contaminated Single Predictor** | **Contaminated Two Predictors** |
| **AASK** | 0.192 | 0.435 | 0.550 | 0.198 | 0.198 | 0.196 | 0.196 |
| **AGES** | 0.122 | 0.298 | 0.378 | 0.126 | 0.127 | 0.124 | 0.124 |
| **ALTOLD** | 0.139 | 0.167 | 0.243 | 0.143 | 0.143 | 0.140 | 0.141 |
| **Onco-GFR** | 0.184 | 0.213 | 0.292 | 0.185 | 0.186 | 0.186 | 0.185 |
| **MDRD** | 0.157 | 0.179 | 0.288 | 0.165 | 0.165 | 0.160 | 0.160 |
| **MESA** | 0.159 | 0.247 | 0.436 | 0.158 | 0.159 | 0.156 | 0.156 |
| **Pakistan** | 0.266 | 0.614 | 0.642 | 0.265 | 0.266 | 0.268 | 0.268 |
| **UMN DONORS** | 0.111 | 0.207 | 0.327 | 0.117 | 0.118 | 0.113 | 0.113 |

RMSE: Root Mean Square Error

Under no added contamination, we fit linear models developed on a random sample of 100 observations from a given study and applied to the remaining observations from that study. We then added mean and variance contamination to a single excellent predictor (pseudouridine alone) or to two excellent predictors (pseudouridine and cystatin-C) and compared to linear models developed and applied within the given study, linear models developed and applied within the given study but *with outlier identification and robust estimation*, and finally transfer learning models *with outlier identification and robust estimation*. Outliers were identified as the two most inconsistent markers and robust prediction was made using transfer learning models with screen predictors. Results are averaged across ten cross-validation iterations.
